# Implementation and Validation of the Roche Light Cycler 480 96-Well Plate Platform as a Real-Time PCR Assay for the Quantitative Detection of Cytomegalovirus (CMV) in Clinical Specimens Using the Luminex MultiCode ASRs System

**DOI:** 10.3390/medsci8010014

**Published:** 2020-03-11

**Authors:** Shengwen Calvin Li, Kara J. Sparks, Leonard S. Sender

**Affiliations:** 1 Neuro-Oncology and Stem Cell Research Laboratory (NSCL), CHOC Children’s Research Institute (CCRI), Children’s Hospital of Orange County (CHOC), 1201 W. La Veta Ave., Orange, CA 92868-3874, USA; 2 Department of Neurology, University of California-Irvine School of Medicine, Orange, CA 92868, USA; 3 Molecular Pathology Laboratory, Bone Marrow Transplantation, Children’s Hospital of Orange County, Orange, CA 92868, USA; 4 Hyundai Cancer Institute, CHOC Children’s Hospital, Orange, The Chao Family Comprehensive Cancer Center, University of California-Irvine School of Medicine, Orange, CA 92868, USA

**Keywords:** cytomegalovirus, infection diseases, PCR, real-time PCR of 96-well plate, Roche Light Cycler 480 PCR device, stem-cell therapies, Luminex MultiCode ASRs System

## Abstract

Allogenic stem-cell therapies benefit patients in the treatment of multiple diseases; however, the side effects of stem-cell therapies (SCT) derived from the concomitant use of immune suppression agents often include triggering infection diseases. Thus, analysis is required to improve the detection of pathogen infections in SCT. We develop a polymerase chain reaction (PCR)-based methodology for the qualitative real-time DNA detection of cytomegalovirus (CMV), with reference to herpes simplex virus types 1 (HSVI), Epstein–Barr virus (EBV), and varicella-zoster virus (VZV) in blood, urine, solid tissues, and cerebrospinal fluid. This real-time PCR of 96-well plate format provides a rapid framework as required by the Food and Drug Administration (FDA) for clinical settings, including the processing of specimens, reagent handling, special safety precautions, quality control criteria and analytical accuracy, precisely reportable range (analyst measurement range), reference range, limit of detection (LOD), analytical specificity established by interference study, and analyte stability. Specifically, we determined the reportable range (analyst measurement range) with the following criteria: CMV copies ≥200 copies/mL; report copy/mL value; CMV copies ≤199 copies/mL; report detected but below quantitative range; CMV copies = 0 with report <200 copies/mL. That is, with reference range, copy numbers (CN) per milliliter (mL) of the LOD were determined by standard curves that correlated Ct value and calibrated standard DNA panels. The three repeats determined that the measuring range was 1E2~1E6 copies/mL. The standard curves show the slopes were within the range −2.99 to −3.65 with *R^2^* ≥ 0.98. High copy (HC) controls were within 0.17–0.18 log differences of DNA copy numbers; (2) low copy (LC) controls were within 0.17–0.18 log differences; (3) LOD was within 0.14–0.15 log differences. As such, we set up a fast, simple, inexpensive, sensitive, and reliable molecular approach for the qualitative detection of CMV pathogens. *Conclusion*: This real-time PCR of the 96-well plate format provides a rapid framework as required by the FDA for clinical settings.

## 1. Introduction

Stem-cell therapy conjures hope for treatment of multiple diseases; however, clinically proven therapies remain elusive due to unpredicted side effects of stem-cell therapies, which root in the heterogeneity of stem cells and the concomitant use of immune suppression agents, triggering infection diseases. As such, traditionally used hematopoietic stem-cell transplantation (HSCT), for example, causes significant morbidity and mortality in allograft recipients, mainly by cytomegalovirus (CMV) infection. In T-cell-depleted transplant or, if severe, acute graft-versus-host disease, ganciclovir prophylaxis or preemptive treatment is applied, even though CMV disease may be as high as 17% in seronegative HSCT recipients. Strategies to prevent late CMV infection and disease are desperately needed, owing to the continued clinical significance of CMV in the HSCT setting, demanding new and more effective anti-CMV drugs [1], as well as owing to current antiviral agents associated with deadly toxicities [2]. All of these require thorough cellular and mechanistic molecular studies of CMV pathogenesis and pharmacological reactions.

Learning from the prophylactic approaches to herpes viruses such as herpes simplex and varicella-zoster, a series of phase III trials for CMV infection in the setting of HSCT shifted toward cellular immunotherapy strategies from those generally involving pre-emptive therapy based on sensitive molecular surveillance. Such adaptive immunity controls CMV infection through the role of natural killer (NK) cells and gamma–delta T cells [3]. The 2015 Tandem American Society underlined the adaptive immunity approach for Blood and Marrow Transplantation/Center for International Blood and Marrow Transplant Meetings, which signals the development of CMV early detection technology.

As CMV derived from HSCT and from human immunodeficiency virus (HIV)-infected patients represents a life-threatening pathogen in patients, quantitative and sensitive detection methods for CMV can help predicting the patient’s risk for disease and in monitoring the effect of antiviral therapy. Current quantitative and sensitive detection methods include viral culture techniques, antigen detection assays, and DNA detection assays (PCR, branched-DNA assay, and the DNA hybrid capture assay). All of these methods gravitated toward the approximate correlation of systemic (more accurate with anatomic sites/site-specific measurement) CMV load and CMV disease activity dynamically related to immunosuppressive therapy, antiviral treatment, and drug resistance. In this way, quantitative CMV detection techniques to direct and monitor antiviral treatment should observe the reproducibility of test results and better standardization of the assays [4]. Real-time (RT) polymerase chain reaction (PCR) offers a rapid, reproducible, and sensitive evaluation of CMV pathogens in the course of diagnostic routine within clinical outcomes, such as neurological disorders and neuropsychiatric symptoms [5], acute exacerbations of ulcerative colitis (UC) [6], uncontrolled plasma HIV viremia and CMV, Epstein–Barr virus (EBV), and HHV-8 (human herpesvirus 8) shedding [7], and detection of hepatic antibodies to antigens of cytomegalovirus (immunoglobulin M (IgM) CMV, IgG CMV, avidity of IgG CMV) in blood and urine [8].

All the above clinical conditions and strategies demand a rapid, sensitive, accurate, and specific detection platform for CMV. The real-time quantitative CMV DNA-specific PCR analysis was used as a surveillance method of bronchoalveolar lavage fluid [9]. The proprietary system Luminex Real-Time PCR Assays are used for detecting Epstein–Barr virus (EBV) [10], herpes simplex viruses (HSV) [11], and hepatitis C virus (HCV) [12]; however, the Luminex Real-Time PCR Assays for detecting CMV remain to be evaluated. Referring to the known criteria from the “CMV Working Group of the Complications of HIV Disease RAC (retinal artery calibers), AIDS (Acquired immunodeficiency syndrome) Clinical Trials Group, and comparison of quantitative and qualitative PCR assays for cytomegalovirus DNA in plasma” [13], we integrated the duplex quantitative Real-Time PCR Assay of the COBAS Amplicor CMV [14] and viral load correlation [15], to design the Roche LightCycler 2 (LC2)-based quantitative PCR and Roche Light Cycler 480 (LC480) 96-well Plate Platforms for detection of cytomegalovirus in blood, urine, and respiratory samples. Here, we describe how the CMV DNA threshold is determined at different levels appropriate in settings of high or low CMV prevalence of predictive values of CMV diseases.

## 2. Materials and Methods

We provide a commercially available polymerase chain reaction (PCR)-based methodology (refer to Section 2.1) on the qualitative real-time DNA detection of cytomegalovirus (CMV), with reference to herpes simplex virus types 1 (HSVI), Epstein–Barr virus (EBV), and varicella-zoster virus (VZV) in blood, urine, solid tissues, and cerebrospinal fluid (refer to Section 2.2). We adhered to good clinical practices (GCPs) by the Food and Drug Administration (FDA) and the Association of Clinical Research Professionals (ACRP) to process all the clinical specimens, as approved by the research compliance agency.

### 2.1. Materials and Reagents

The MultiCode^®^-RTx DNA Reagent Set and ASRs were produced by Luminex Corporation (Austin, TX, USA). Other materials and suppliers were described previously [16]. To develop and optimize the analytic performance characteristics, we purchased CMV with reference materials such as viral standards, controls, and WHO (The World Health Organization) Samples (SeraCare Life Sciences, Milford, MA, USA; ZeptoMetrix Corp., Franklin, MA, USA) as previously described [11], including specificity, quantitative linearity, lower limits of detection (LLODs) and quantification (LLOQs), analytical measurement range, and quantitative variability of results. Validation of the standard operating procedure for this study was performed by using the de-identified aliquots of the leftover clinical specimens collected between 2010 and 2012 for molecular testing, which were obtained with IRB approval from the Children’s Hospital of Orange County (Number: MOL503v1, December 12, 2009). The procedure was adhered to the College of American Pathologists (CAP), the leading organization of board-certified pathologists, and the CAP-accredited laboratory for virus testing of samples (excluding purified DNA/RNA, FFPE (formalin fixed paraffin embedded), or urine), including those that must be negative for hepatitis B surface antigen, hepatitis C antibody, and HIV I and II antibodies. The CAP-certified standards were accordingly obtained for control references.

### 2.2. Experimental Procedures

Specimen DNA extraction for viral pathogen materials and human tissues was as previously described [17]. The extraction of viral RNA and the storage of sample material were critical, as viral RNA integrity affected qPCR sensitivity and specificity. Viral DNA was prepared by following the viral nucleic acid extraction manual (Qiagen, Valencia, CA, USA). DNA samples were derived from blood, plasma, serum, urine, cerebrospinal fluid (CSF), and respiratory samples. The blood, plasma, serum, and urine specimens were validated as guided by federal, state, and institutional review board regulations. All patient specimens were used with de-identifiers for testing. Special safety precautions of standard protocols of the clinical laboratory were observed.

### 2.3. PCR Amplification and Detection

The Roche Light Cycler 480, a real-time PCR instrument, was programmed with a thermal cycling profile (Table 3) before making the reaction mixtures (Table 1 and Table 2)). The reaction vessel(s) was pre-chilled in a cold block placed at 4 °C before the reaction was set up. All reagents provided with the MultiCode^®^-RTx DNA Reagent Set (Table 1) were completely thawed at room temperature (20 to 25 °C). Once thawed, all reagents were briefly vortexed, including TITANIUM *Taq* DNA polymerase, followed by briefly spinning the centrifuge tubes to ensure maximum recovery of the contents. All reagents were on ice or cold blocks. Appropriate target materials were used for the DNA template. The target material must be suitable for DNA qPCR. The extraction used must result in high-quality DNA that is free of inhibitors for reproducible results (note results with reference materials (SeraCare Life Sciences, Milford, MA, USA; ZeptoMetrix Corp., Franklin, MA, USA), as described previously [11]). Negative control material was used in triplicates in every run to monitor contamination by replacing 5 μL of DNA template with nuclease-free water. The MultiCode^®^-RTx DNA Reagent Set (Luminex Corporation, Austin, TX containing sufficient material for 100 reactions of 25 μL was used to fill up a 96-well PCR reaction plate. The recommended reaction set-up consisted of 20 μL of master mix and 5 μL of the target DNA. Increased reaction volumes and target volumes can be used if the master mix preparation was adjusted appropriately. The master mix example in Table 1 assumed that target primers (which came with the reagent kit) and control primers were prepared at 25× concentration. The recommended final concentration for each primer was 100–400 nM. Multiple target primers might be combined in a master mix by substituting a portion of the nuclease-free water for the primers. The total volume of the master mix per reaction was 20 μL. The flow of the procedure started with the preparation of the master mix in Table 1 with DNase-free aerosol barrier pipette tips. Combined reagents were kept in a clean microcentrifuge tube on ice or cold blocks. All reagents were added in the order listed to ensure sufficient material for the number of reactions being performed, including overage volume.

The above master mix was mixed well with vortex and centrifuged briefly. Then, 20 μL of the master mix was added to each reaction vessel in Table 2, followed by adding five μL of the target DNA to each reaction vessel or well (96-well plate) in Table 2; the mixture of the reactions was kept on ice-cold block throughout this procedure. The amplification mixture that used Luminex ASR, using labeled primer chemistries with synthetic nucleotide base pairing, was performed in a total volume of 25 μL that contained 5 μL of 5× ISOlution, 1 μL of each of the CMV primer pair, Internal Control Primers 3 and DNA universal sample processing control, 0.5 μL of Titanium Taq (Clontech Laboratories, Mountain View, CA, USA), 5 μL of sample, and nuclease-free water to bring the total to 25 μL (Table 1 and Table 2).

The 96-well plate was briefly centrifuged using plate centrifugation or by centrifuging the reaction vessel(s) before placing them in the real-time PCR instrument. The instructions supplied with the real-time PCR instrument Roche Light Cycler 480 for appropriate operation and maintenance procedures were referenced for the thermal cycling parameters, as listed in Table 3. For further details about programming the cycling profile, PCR reaction conditions were detailed previously [16].

### 2.4. Data Analysis

At the end of the run, the data were exported according to the data analysis software using the MultiCode^®^-RTx analysis software (EraGen Biosciences, Inc., Madison, Wis., USA). Luminex results were analyzed with MultiCode^®^-RTx analysis software version 1.6.2.10 (Luminex Corporation, Austin, TX, USA). All statistics were determined, as described previously [18].

## 3. Results

**Figure 1:**
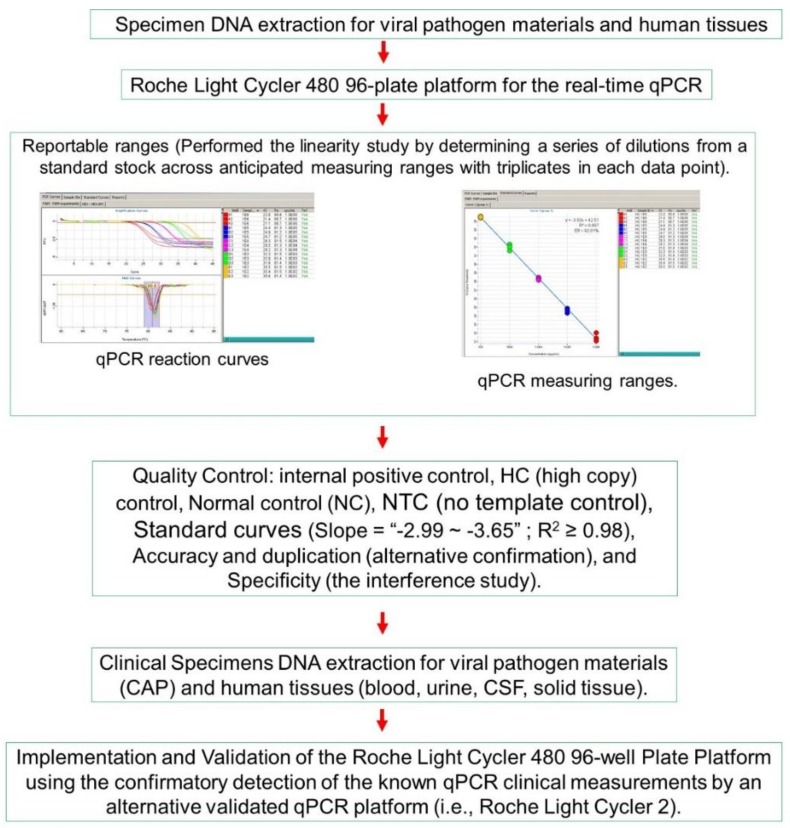
Overall schematic diagram representing the workflow.

It is well known that FDA-approved and cleared assays in molecular methods and platforms for infectious disease testing must meet certain standards with specific guidelines [19]. We followed the guidelines of Burd’s article [18]. This article articulates the “laboratory-developed tests” for molecular diagnostics, providing guidelines by following the regulations issued by the Clinical Laboratory Improvement Amendments (CLIA) and cleared or approved by the FDA. The specific steps are outlined in Figure 1.

### 3.1. Analytical Performance

#### 3.1.1. Specimens in Compliance

DNA sample integrity derived from blood, plasma, serum, urine, cerebrospinal fluid (CSF), and respiratory samples were determined by agarose gel electrophoresis, and the quantity was quantified using a NanoDrop™ 2000/2000c UV–Vis spectrophotometer (Thermo Scientific™). The collection procedures for blood, plasma, serum, and urine specimens were validated as guided by following federal, state, and institutional review board regulations. All patient’s specimens were used with de-identifiers for testing. Special safety precautions of standard protocols of the clinical laboratory were observed.

#### 3.1.2. Reportable Range: The Linearity Study

Firstly, we determined the reportable range and performed the linearity study by establishing a series of dilutions from a standard stock across the anticipated measuring range with triplicates in each data point (Figure S1, standard curve 1; Figure S2, standard curve 2; Figure S3, standard curve 3). Secondly, we obtained all the required parameters, including the following: (i) the linearity study was determined with three repeats, and the measuring range was 1E2~1E6 copies/mL (Refer to Table S4.0.1. Overall Log difference in the limit of detection (LOD) variations between standard curves); (ii) the IPC (internal positive control) showed the amplification for the IPC within normal limits; (iii) the normal control (NC) had the amplification for IPC, but not CMV; (iv) the NTC (no template control) had no amplification for either IPC or CMV; (v) three independent standard curves created by using three separated DNA extractions of the known ZeptoMetrix-calibrated samples showed that the slopes of these standard curves were within the range −2.99 to −3.65 with *R^2^* ≥ 0.98 (Figures S1–3). Based on these curves, we found that the HC control was within 0.5 logs of 1E5 (Table S4.0.2. Overall log difference in the high copy (HC) variations between standard curves); the LC control was within 0.5 logs of 400 copies/mL (Table S4.0.3. Overall, Log difference in the low copy (LC) variations between standard curves). Specifically, (i) the HC control was within 0.17–0.18 log differences of DNA copy numbers, (ii) the LC control was within 0.17–0.18 log differences (Table S4.1.3), and (iii) the LOD was within 0.14–0.15 log differences (Table S4.1.1 LOD was determined by replication experiments performed on different dates (CN—copy number per mL; LOD—limit of detection; SD—standard deviation).

### 3.2. Sensitivity

The limit of detection (LOD) values were determined on three different days using 40 triplicates for each concentration of viral samples. In all three experiments, consistent results were obtained, as shown in Figure S4. Quantitative real-time PCR of 50 patients’ viral DNA was run side-by-side with calibrated DNA standard reagents for creating standard curve 1 with controls of NC, NTC (water), and IPC within the same 96-well plate for intra-assay comparison. Multiple lines of independently controlled experiments showed consistent results (Figure S4; Tables S4, Table S5, Table S6). We determined analytic sensitivity that was measured by analytical LOD (limit of detection). Specifically, LOD/analytical sensitivity was firstly determined by titrating the known copy numbers of 400, 200, and 100 copies/mL, with a blank control (no template control, NTC). When the lowest copy number (i.e., the lowest LOD, LLOD) was detected, then 40 replicates of the LLOD were tested to determine if the LLOD could be detected ≥95% of the time to verify the limit of detection. This sensitivity was further verified by an interference study of mixing CMV-positive samples with EBV-, BK-, or HSV-positive samples to verify that co-infection did not affect lower-end sensitivity. As acceptable criteria, all results had to correlate within 0.5 log difference from the stated concentration. Mixed samples had to retain values within a 0.5 log difference. As shown in Table S4.1, in all experiments performed, 0.12–0.13 (calculated SD) of the log differences of DNA copy numbers (CN) per mL for LLOD were obtained within a run, day to day, in terms of total variation, which was below the 0.5 log difference, as the standard recommended). Furthermore. 74.3 ± 28.6 copies/mL in 40 replicates on different dates were determined for the 100 copies/mL calibrated samples 100% of the time.

### 3.3. Precision Determination by Replication Experiments

**Figure 2:**
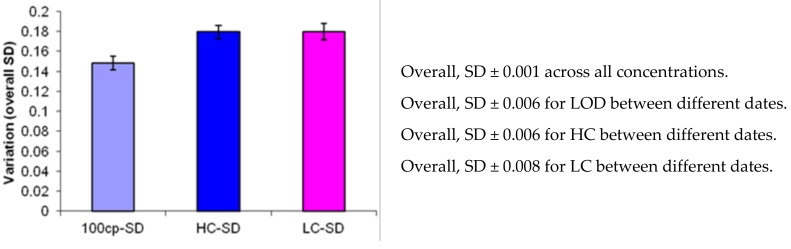
The precision of the LC480 PCR system. The overall variation is summarized from the data of Table S4.0.1; Table S4.0.2; Table S4.0.3; Table S4.1.1; Table S4.1.2; Table S4.1.3; Table S4.2.1; Table S4.2.2.; Table S4.2.3.). The overall variation of log differences across three independent standard curves was SD ± 0.001 across all concentrations of more than 40 replicates (LOD 100 copies/mL; HC (high copies) 1E5 copies/mL; LC (low copies) 400 copies/mL). The overall variation of log differences across three independent standard curves was SD ± 0.006 for LOD across different dates. The overall variation of log differences across three independent standard curves was SD ± 0.006 for HC across different times. The overall variation of log differences across three independent standard curves was SD ± 0.008 for LC across different dates. Note that all these log differences are below the set value of log 0.5, indicating that the assay is consistent within runs, between runs, and between different days. Analyte stability analyses indicated that analytes at various times showed log differences of the limit of detection (LOD) in 100 copies/mL within 0.12–0.14, which reflected the value of 74.3 ± 28.6 copies/mL in 40 replicates on different dates based on the 100 copies/mL calibrated samples. HC showed 0.12–0.14 log differences; LC showed 0.11–0.16 log differences. All of these differences were significantly below the set criterion of 0.5 log differences (see Section 2.4 for details).

In all the clinical molecular assays, precision is essential to validate a system. The precision is determined by replication experiments, including day-to-day, run-to-run, person-to-person, log-to-log, and place-to-place variation. Our datasets indicated that the overall variation of log differences across three independent standard curves was SD ± 0.001 across all concentrations of more than 40 replicates (LOD 100 copies/mL; HC 1E5 copies/mL; LC 400 copies/mL) (Figure 2). The overall variation of log differences across three independent standard curves was SD ± 0.006 for LOD across different dates (Tables S4.0.1 and S4.1.1). The overall variation of log differences across three independent standard curves was SD ± 0.006 for HC across different times (Table S4.0.2. The overall variation of log differences across three independent standard curves was SD ± 0.008 for LC across different dates (Table S4.0.3). Note that all these log differences are below the set value of log 0.5, indicating that the assay was consistent within runs, between runs, and between different days. Analyte stability analyses indicated that analytes at various times showed log differences of the limit of detection (LOD) in 100 copies/mL within 0.12–0.14, which reflected the value of 74.3 ± 28.6 copies/mL in 40 replicates on different dates based on the 100 copies/mL calibrated samples. HC showed 0.12–0.14 log differences; LC showed 0.11–0.16 log differences. All of these differences were significantly below the set criterion of 0.5 log differences. Taken together, these parameters indicated that our LC480 PCR assay system met the standard, as set by Burd’s guidelines [18].

Precision was determined using replication experiments with a minimum of three concentrations (high, low, LOD) tested in duplicate 1–2 times/day; SD was calculated within runs, between runs, and day to day, as well as in terms of the total variation. The experimental criteria were as follows: (i) each sample was tested three times; (ii) one assay was tested on two different days by the same technician; (iii) two different technicians tested the same assay; (iv) acceptance criteria set for all results had to correlate within 0.5 log differences. In summary, we obtained the results shown in Tables S4.1 and S4.2. In all experiments performed, 0.14–0.18 log differences of DNA copy numbers (CN) were obtained within a run, day to day, and in terms of the total variation, which was below the 0.5 log difference recommended by the standard.

### 3.4. Specificity

Analytical specificity was firstly determined using different control virus sample studies of CMV, BK, EBV, EVII, HSVI, and *Bordetella* bacterial DNA, followed by virus interference studies (see Table 4). All these experiments showed that CMV primers specifically detected CMV, with no detection of any other interference viruses or bacterial DNA.

### 3.5. Accuracy

Accuracy (trueness) was also evaluated as supported by following FDA guidelines and manual. The standard calibrated panels and patients’ samples were determined for accuracies side-by-side with multiple lines of controls (positives, negatives, and blanks). All the patient specimens were validated with an alternative platform based on Roche ASRs Light Cycler 2.0 in our laboratory).

In summary, we found that CMV primers specifically detected CMV, not any of the other viruses tested. Secondly, no interference was detected between the mixtures of CMV and any other viruses tested, as well as controls with CMV alone, as shown in the copy number log difference of 0.007 (0.081 vs. 0.088), which is significantly lower than 0.5 log difference of the set criterion. Thirdly, the patient urine samples were determined successfully with the LC480 96-well platform; however, it seemed that the LC480 platform was more sensible than the LC2 platform. Specifically, LC480 showed higher copy numbers than the LC2 platform, which may be further determined with a larger sample size.

### 3.6. Qualitative Performance Using Clinical Samples

After we defined the parameters that satisfied the FDA standards (above), we attempted to test the LC480 system in clinical specimens. As shown in Figure S4.2.4., we performed quantitative real-time PCR of 50 patients’ viral DNA, which was run side-by-side with calibrated DNA standard reagents for creating standard curve 1 (1E6, 1E5, 1E4, 1E3, 1E2) with controls of LC (low copy: LCA, LCA-B)), CAP (VLS9, V3, V4), NTC (water), IPC (HEX) within the same 96-well plate for intra-assay comparison. The data met the requirements as set for clinical detection.

## 4. Discussion

Our principal finding during CMV PCR detection was related to the quality control (QC) of the implementation procedure. Such QC criteria included (i) an IPC (internal positive control) with amplification for the IPC within normal limits (Ct = 33.5/Tm = 78.5 ± 1 °C), (ii) an HC (high copy) control within 0.5 log of 1e5 with Tm values 80.1 ± 1 °C, (iii) an LC (low copy) control within 0.5 log of 400 copies/mL with Tm values 80.1 ± 1 °C, (iv) a normal control (NC) with amplification for IPC, but not CMV, (v) an NTC (no template control) with no amplification for either IPC or CMV, (vi) a standard curve with slope of −2.99 to −3.65 *R^2^* ≥ 0.98, (vii) sensitivity, accuracy, and duplication, with more than 40 replicates of the previously tested samples tested for accuracy, and (viii) specificity, which was related to the specific detection of CMV. Such QC criteria helped to obtain valid results. The data showed that all QC criteria and analytic accuracy were strictly observed. Specifically, 100% accuracy for NC, NTC, positive, and negative controls was obtained. Furthermore, all three standard curves showed a slope of −2.99 to −3.65, *R^2^* ≥ 0.98. Thirdly, quantitative results showed that (a) the HC control was within 0.17–0.18 log differences of DNA copy numbers, (b) the LC control was within 0.17–0.18 log differences, and (c) the LOD was within 0.14–0.15 log differences. All values were obtained with Tm values 80.1 ± 1 °C. We determined the reportable range (analyst measurement range) with the following criteria: CMV copies ≥200 copies/mL; report copy/mL value: CMV copies ≤199 copies/mL; report detected but below quantitative range; CMV copies = 0 with report <200 copies/mL. That is, with the reference range, copy numbers (CN) per milliliter (mL) of the LOD were determined by the standard curves that correlated Ct value and calibrated standard DNA panels. The three repeats determined that the measuring range was 1E2~1E6 copies/mL. The standard curves showed slopes within the range −2.99 to −3.65 with *R^2^* ≥ 0.98. HC controls were within 0.17–0.18 log differences of DNA copy numbers, LC controls were within 0.17–0.18 log differences, and LOD was within 0.14–0.15 log differences.

We got through some common myths in performing qPCR; for example, we found that clinical specimens stored at 4 °C were stabilized for RNA integrity for extraction up to three months, and isolated genomic DNA was preserved for up to one year at 4 °C, similarly to what was reported by Bishop’s group [20]. Consistent with Bishop’s finding, we found that the use of unstable internal control reference genes leads to substantial differences in the conclusive results. Bishop’s colleagues also stated that cDNA content can be used for data normalization; however, complete removal of RNA from cDNA samples is essential for obtaining accurate cDNA content. We think that the internal control is essential for qPCR in clinical testing, which is consistent with another previous report [21]. Nevertheless, the whole-genome sequencing (WGS) of viral pathogens demands the highly stringent extraction of viral RNA and the storage of sample materials, as PCR-based detection methods focus on small amplicons, and viral WGS applications require RNA of high quality and integrity for adequate sequence coverage and depth [22]. Ultimately, the integrity of viral DNA/RNA preparation from viral pathogens needs to measure up with the comparability of CMV assays with the first WHO International Standard for human CMV [23]. In the standard dilutions, their tests correlate well with the standard curve (*R^2^* > 0.96), showing good agreement for whole blood (*R^2^* = 0.79), as well as for other specimen types (*R^2^* = 0.93). In our report, we obtained *R^2^* = 0.990–0.997.

Analytical specificity, being the most significant element in an assay, must be established by an interference study, as described previously [11]. We performed the assays side-by-side with sample-related interfering substances and genetically similar organisms or organisms found in the same sample sites with the same clinical presentation of CMV, including mixtures of CMV and other viruses, with distinct viruses alone as controls to assess the specificity (i.e., a low concentration of the analyte (CMV) was mixed with either EBV or BK). Acceptable criteria included the following: (1) all results had to correlate within 0.5 log difference of the stated concentration; (2) mixed samples had to retain a value within 0.5 log difference. Our results showed that CMV primers specifically detected CMV, but not any of the other viruses tested. Additionally, no interference was detected between the mixtures of CMV and any other viruses tested, as well as controls with CMV alone, as shown in the copy number log difference of 0.007 (0.081 vs. 0.088), which is significantly lower than the 0.5 log difference of the set criterion. Lastly, the patient urine samples were determined successfully with the LC480 96-well platform; however, it seemed that the LC480 platform was more sensible than the LC2 platform. Specifically, LC480 showed higher copy numbers than the LC2 platform, which may be further determined with a larger sample size. These two platforms provide advantages over the earlier versions of qPCR detection for human cytomegalovirus [24], with TaqMan probes and molecular beacons [25], as well as in immunosuppressed pediatric patients [26].

In conclusion, this real-time PCR of the 96-well plate format provides a rapid framework as required by the FDA for clinical settings.

## Figures and Tables

**Table 1:** MultiCode-RTx qPCR DNA reagent master mix.

	Compositions	1 Target per Reaction (μL)
1	Nuclease Free Water, 2 mL (PN 1468)	11
2	5× ISOlution, DNA Use, 500 μL (PN 1790) Lot 1130404	5
3	CMV Primers (PN 3906) Lot 1204003	1
4	25× Control Primers 3 (PN 3804), Lot 1204002	1
5	DNA Universal Reference, 100 μL (PN 1844), Lot 1114317	1
6	UNG (Uracil-DNA-N-Glycosylase) (No lot #) Roche_12790500 Sept 2012	0.5
7	TITANIUM™ Taq DNA polymerase (50x), S1792 Lot 1003099, (Clontech Cat. No. 639208 or 639272)	0.5
	Total volume	20

**Table 2:** Reaction volume with target and master mix.

Compositions	Volume (μL)
Master mix (see Table 1)	20
Target DNA	5
Total volume	25

**Table 3:** Thermal cycling parameters.

			Temperature	Control		
Program	Cycles	Target (°C)	Acquisition Mode	Hold (hh:mm:ss)	Ramp Rate (°C/s)	Analysis Mode
UNG	1	40	None	0:10:00	4.4	None
Denaturation	1	95	None	0:10:00	4.4	None
PCR	45	95	None	0:00:05	4.4	Quantification
		58	None	0:00:10	2.2	
		72	Single	0:00:20	1	
Melt	1	95	None	0:00:10	4.4	Melting Curves
		60	None	0:00:30	2.2	
		95	Continuous			
Melt	1	40	None	0:00:30	2.2	Yes

**Table 4:** Analytical specificity as determined by the interference study of cytomegalovirus (CMV) with BK virus, Epstein–Barr virus (EBV), herpes simplex virus type I (HSVI), and varicella-zoster virus (VZV). N/A—not applicable; NC—normal control; NTC—no template control.

	Sample ID	Ct	Tm	Roche-LC480 Platform (Copy/mL)	Log Copy Number
6/13/2012	2012vsID1-01_VZV (5 μL)	N/A	N/A	N/A	
Stnd3	2012vsID1-04_EBV (5 μL)	N/A	N/A	N/A	
	2012vsID1-05_HSVI (5 μL)	N/A	N/A	N/A	
	CAP2011-VLS01_BK (5 μL)	N/A	N/A	N/A	
	NC	N/A	N/A	N/A	
	ID1-01+CMV5E5 (1:1–2.5 μL:2.5 μL)	22	81	2.00E+05	5.30103
	ID1-04+CMV5E5 (1:1–2.5 μL:2.5 μL)	23	80.9	1.70E+05	5.230449
	ID1-05+CMV5E5(1:1–2.5 μL:2.5 μL)	22	80.5	2.40E+05	5.380211
	CAP2011-VLS01+CMV5E5(1:1–2.5 μL:2.5 μL)	22	81.1	3.10E+05	5.491362
	mean			230000	5.350763
	SD			60553.01	0.111929
	NTC	N/A	N/A	N/A	
	CMV5.00E+05(5 μL)	21	81.1	6.90E+05	5.838849
	CMV5.00E+05(5 μL)	21	81.1	5.10E+05	5.70757
	CMV5.00E+05(5 μL)	22	81.1	2.60E+05	5.414973
	CMV5.00E+05(5 μL)	22	81.2	3.50E+05	5.544068
	mean			452500	5.626365
	SD			189098.7	0.185475
	NC	N/A	N/A	N/A	N/A

Note: College of American Pathologists (CAP) samples were as follows: “2012 viral survey ID1-01_VZV” was for the varicella-zoster virus. “2012 viral survey ID1-04_EBV” was positive for Epstein–Barr virus. “2012vsID1-05_HSVI” was positive for herpes simplex type I virus. “CAP2011-VLS01_BK” was positive for BK viral load. The procedure and table design adhered to the College of American Pathologists (CAP), the leading organization of board-certified pathologists, and the CAP-accredited laboratory for virus testing of samples. BK: BK virus is an abbreviation of the name of the first patient whom the virus was isolated from; BK virus (BKV) belongs to the human Polyomaviridae, initially isolated from urine sample of a 29-year-old male patient with renal blockage and failure at 1971, which is also called polyomavirus.

## Supplementary Materials

The following are available online at https://www.mdpi.com/2076-3271/8/1/14/s1.

## Abbreviations

BK (a member of the polyomavirus family, the BK virus was first isolated in 1971 from the urine of a renal transplant patient, initials B.K.), CAP (College of American Pathologists), CMV (cytomegalovirus), CSF (cerebrospinal fluid), EBV (Epstein–Barr virus), GCP (good clinical practices), HCV (hepatitis C virus), HSV I/II (herpes simplex virus types 1 and 2), LC2 (Roche LightCycler 2-based quantitative PCR), LC480 (Roche Light Cycler 480 96-well plate platform), LLOD (lower limit of detection), LLOQ (lower limit of quantification, PCR (polymerase chain reaction), VZV (varicella-zoster virus).
