# Supplementary material for: Implementation and Validation of the Roche Light Cycler 480 96-Well Plate Platform as a Real-Time PCR Assay for the Quantitative Detection of Cytomegalovirus (CMV) in Clinical Specimens Using the Luminex MultiCode ASRs System
Source: Med Sci (Basel). 2020 Mar 11;8(1):14. doi: 10.3390/medsci8010014 (PMC7151591; doi:10.3390/medsci8010014)
Supplement: Supplementary file 1 [file medsci-08-00014-s001.pdf]

Supplementary Materials

Implementation and Validation of the Roche Light Cycler 480 96-Well Plate Platform as a Real-Time PCR Assay for the Quantitative Detection of Cytomegalovirus (CMV) in Clinical Specimens Using the Luminex MultiCode ASRs System

Shengwen Calvin Li [1,2,*], Kara J. Sparks [3] and Leonard S. Sender [4]

1 Neuro-Oncology and Stem Cell Research Laboratory (NSCL), CHOC Children's Research Institute (CCRI), Children's Hospital of Orange County (CHOC), 1201 W. La Veta Ave., Orange, CA 92868-3874, USA
2 Department of Neurology, University of California-Irvine School of Medicine, Orange, CA 92868, USA
3 Molecular Pathology Laboratory, Bone Marrow Transplantation, Children's Hospital of Orange County, Orange, CA 92868, USA; karajsparks@gmail.com
4 Hyundai Cancer Institute, CHOC Children's Hospital, Orange, The Chao Family Comprehensive Cancer Center, University of California-Irvine School of Medicine, Orange, CA 92868, USA; leonardsender@gmail.com
* Correspondence: shengwel@uci.edu; Tel.: +714-509-4964

Received: 17 January 2020; Accepted: 7 March 2020; Published: date

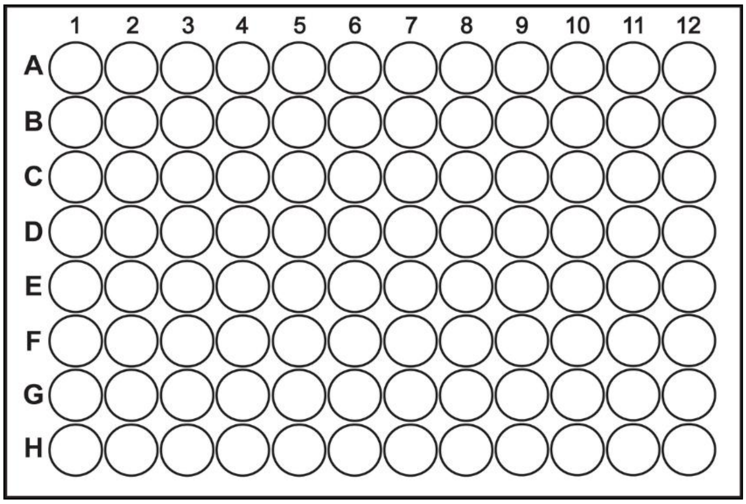

Notes for All the supplemental Figures and Tables: PCR plate grid template shows the location (as known as "Well" in the plate) is labeled as: A1, A2, …, A12; B1 – B12, which is shown on all the supplmental Tables (as known as Location in Tables) or the original PCR profiles (as known as Wells) below.

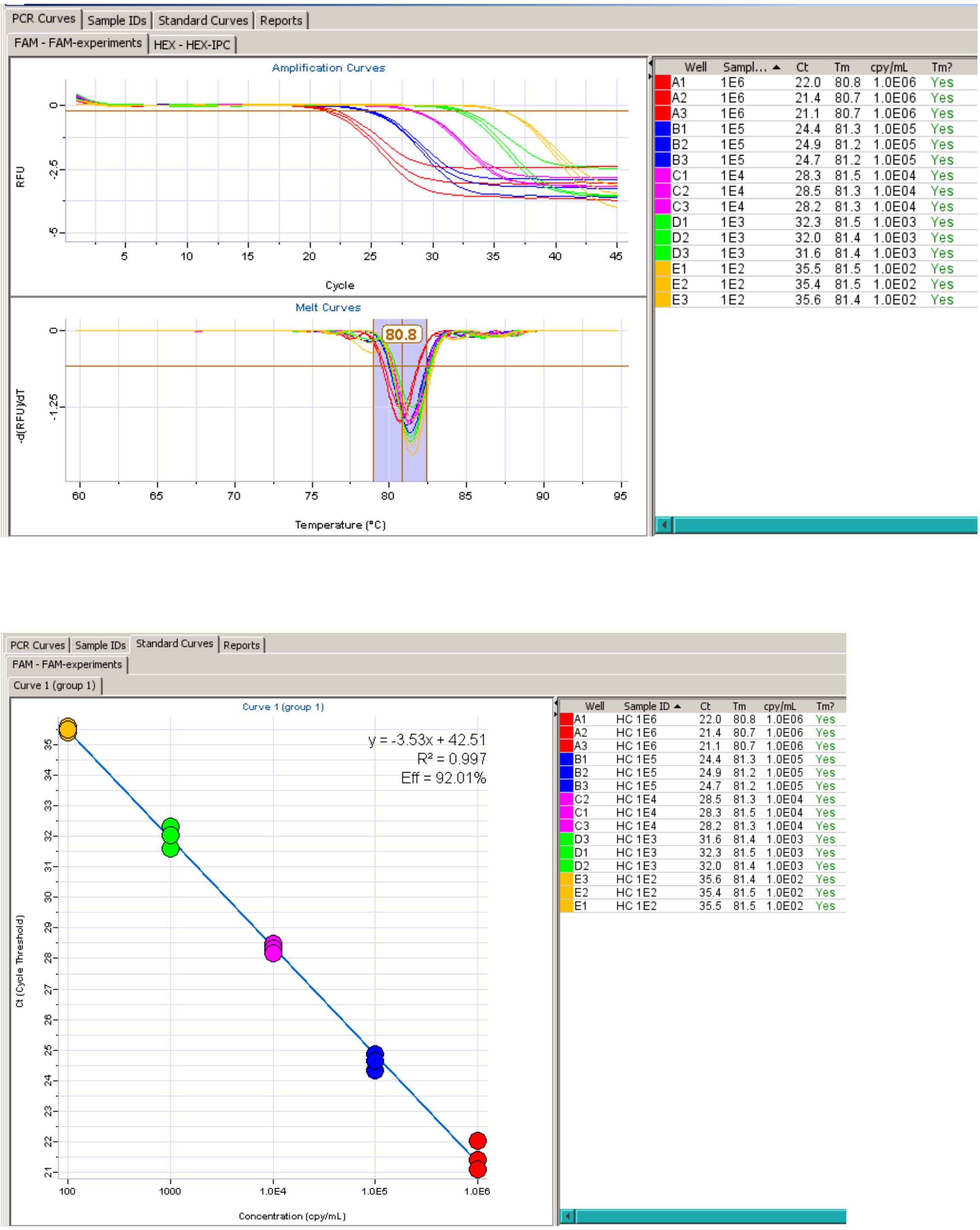

Figure S1. LC480's original PCR profiles show the Reportable range. We performed the linearity study by determining a series of dilutions from a standard stock across anticipated measuring range with triplicates in each data point (performed on March 21, 2011). Upper panel: qPCR reaction curve; lower panel: measuring range. Note: Plot of results from a linearity experiment to determine reportable range. Seven concentrations of analyte prepared by dilution of a high-concentration standard were tested in triplicate. Assigned values, (converted to $\log_{10}$) were plotted on the x axis versus measured values (converted to $\log_{10}$) on the y axis using Microsoft Excel. Linear regression analysis gave the equation y = -3.53x + 42.51 (R^2 = 0.997) (Reference: Burd EM, 2010).

(abbreviations: LC480, Roche Light Cycler 480 96-well Plate Platform; LC2, Roche Light Cycler 2 microcapillary Platform).

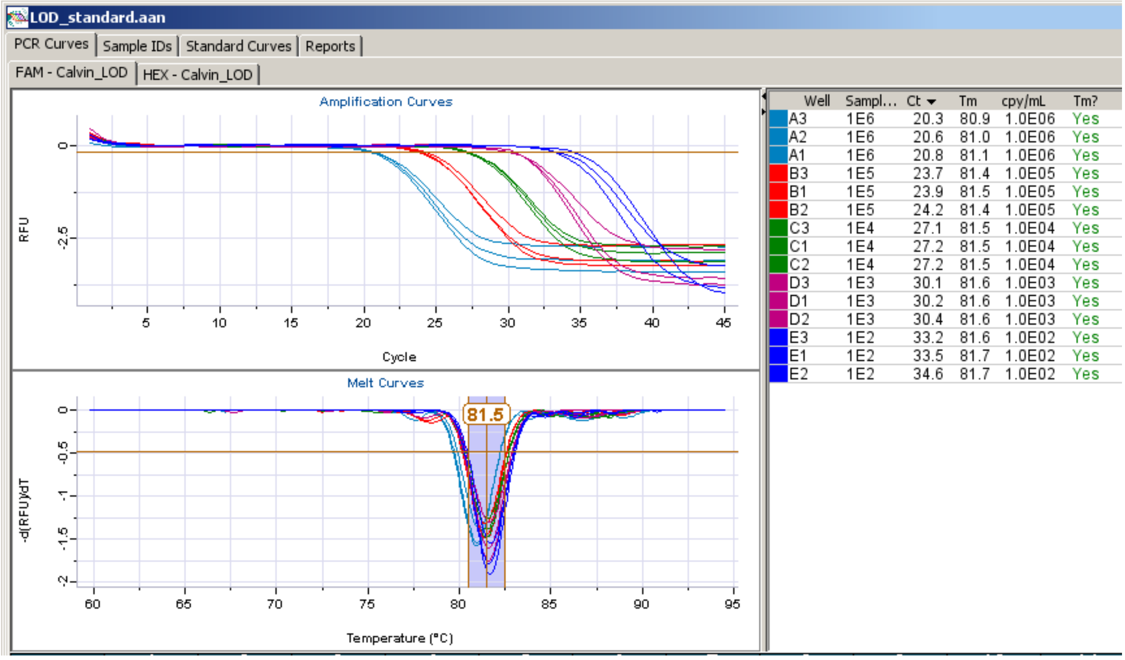

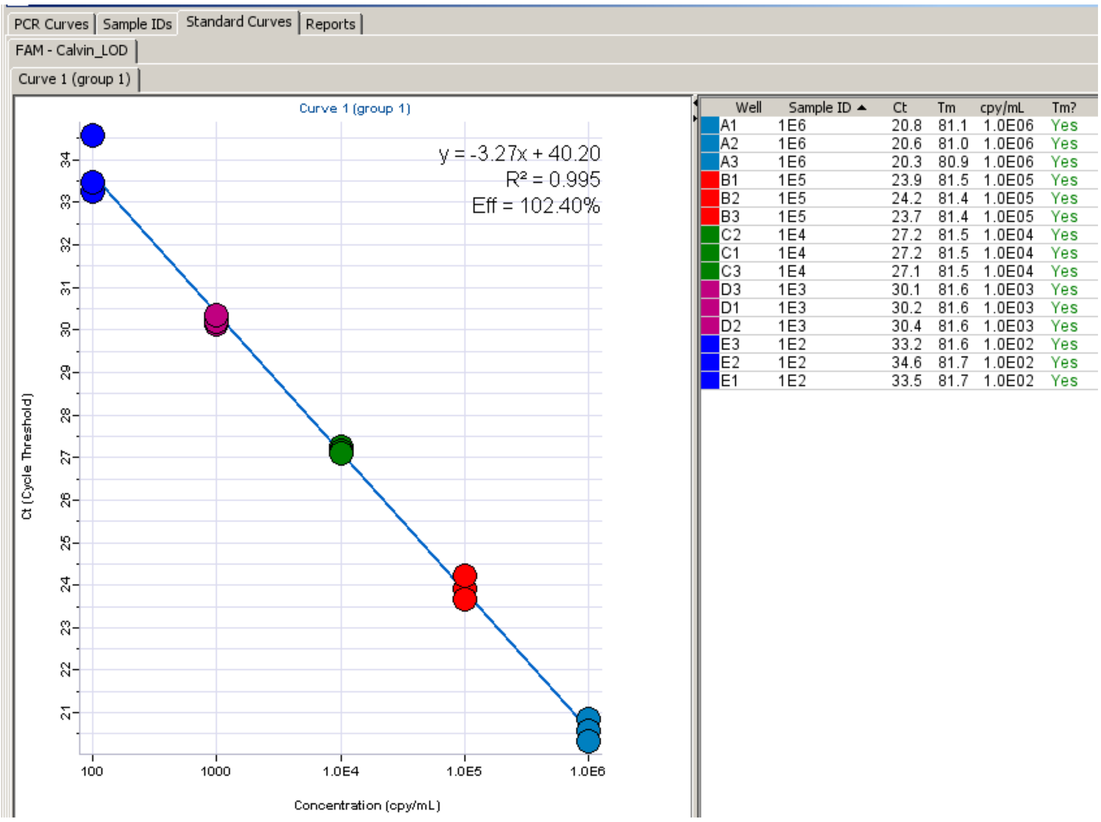

Figure 2. LC480's original PCR profiles show the Reportable range. We performed the linearity study by determining a series of dilutions from a standard stock across anticipated measuring range with triplicates in each data point (performed on April 23, 2011). Upper panel: qPCR reaction curve; lower panel: measuring range. Note: Plot of results from a linearity experiment to determine reportable range. Seven concentrations of analyte prepared by dilution of a high-concentration standard were tested in triplicate. Assigned values, (converted to $\log_{10}$) were plotted on the x axis versus measured values (converted to $\log_{10}$) on the y axis using Microsoft Excel. A second-order polynomial trendline of the linear regression analysis gave the equation "y = -3.27x + 40.20 (R^2 = 0.995)" (Reference: Burd EM, 2010).

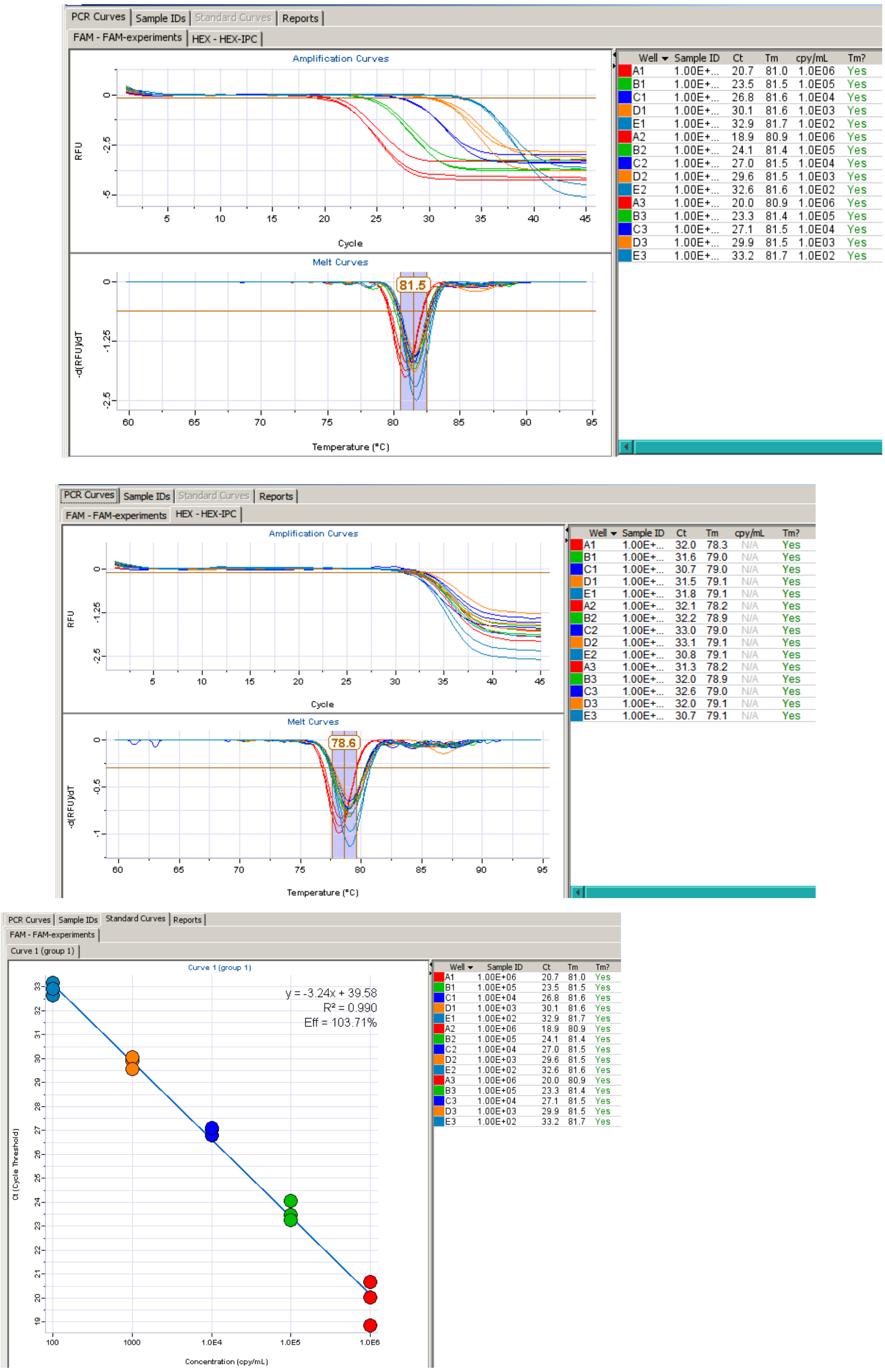

Figure S3. . LC480's original PCR profiles show the Reportable range. We performed the linearity study by determining a series of dilutions from a standard stock across anticipated measuring range with triplicates in each data point (performed on May 24, 2011). Upper panel: qPCR reaction curve; Middle panel: qPCR reaction internal positive control (IPC-HEX); lower panel: measuring range. Note: Plot of results from a linearity experiment to determine reportable range. Seven concentrations of analyte prepared by dilution of a high-concentration standard were tested in triplicate. Assigned values, (converted to $\log_{10}$) were plotted on the x axis versus measured values (converted to $\log_{10}$) on the y axis using Microsoft Excel. A third-order polynomial trendline of the linear regression analysis gave the equation "$y = -3.24x + 40.20$ ($R^2 = 0.990$)" (Reference: Burd EM, 2010).

Table S4.0.1. Overall Log difference in the limit of detection (LOD) variations between standard curves

Stnd1	LOD (100)		stnd2(6/7/12)	LOD (100)		Stnd3	LOD (100)	
Date	CN	Log	Date	CN	Log	Date	CN	Log
**4/23/2012**	1.60E+02	2.20412	**4/23/2012**	5.50E+01	1.740363	**4/23/2012**	3.40E+01	1.531479
	2.00E+02	2.30103		6.60E+01	1.819544		4.10E+01	1.612784
	1.10E+02	2.041393		3.60E+01	1.556303		2.20E+01	1.342423
**4/25/2012**	1.60E+02	2.20412	**4/25/2012**	6.70E+01	1.826075	**4/25/2012**	3.40E+01	1.531479
	1.10E+02	2.041393		6.50E+01	1.812913		3.20E+01	1.50515
	2.10E+02	2.322219		5.50E+01	1.740363		2.00E+01	1.30103
	1.70E+02	2.230449		5.10E+01	1.70757		4.20E+01	1.623249
	2.40E+02	2.380211		3.30E+01	1.518514		4.80E+01	1.681241
	2.80E+02	2.447158		6.80E+01	1.832509		5.90E+01	1.770852
	1.60E+02	2.20412		7.80E+01	1.892095		3.20E+01	1.50515
	2.20E+02	2.342423		9.40E+01	1.973128		4.60E+01	1.662758
	2.20E+02	2.342423		5.20E+01	1.716003		4.40E+01	1.643453
	2.30E+02	2.361728		7.40E+01	1.869232		4.70E+01	1.672098
	2.90E+02	2.462398		7.00E+01	1.845098		6.10E+01	1.78533
	3.10E+02	2.491362		7.60E+01	1.880814		6.50E+01	1.812913
	1.40E+02	2.146128		9.80E+01	1.991226		2.60E+01	1.414973
	1.70E+02	2.230449		1.00E+02	2		3.50E+01	1.544068
	2.00E+02	2.30103		4.30E+01	1.633468		4.00E+01	1.60206
	3.30E+02	2.518514		5.60E+01	1.748188		7.00E+01	1.845098
	1.90E+02	2.278754		6.50E+01	1.812913		3.90E+01	1.591065
	2.60E+02	2.414973		1.10E+02	2.041393		5.40E+01	1.732394
	2.10E+02	2.322219		6.30E+01	1.799341		4.20E+01	1.623249
	2.00E+02	2.30103		8.80E+01	1.944483		4.10E+01	1.612784
	2.10E+02	2.322219		6.90E+01	1.838849		4.30E+01	1.633468
	1.90E+02	2.278754		6.00E+01	1.778151		3.70E+01	1.568202
	1.10E+02	2.041393		3.30E+01	1.518514		2.10E+01	1.322219
**5/24/2012**	2.00E+02	2.30103	**5/24/2012**	7.20E+01	1.857332	**5/24/2012**	4.10E+01	1.612784
100cp14Rep	2.30E+02	2.361728	stnd2(6/7/12)	7.80E+01	1.892095	100cp14Rep	4.80E+01	1.681241
Stnd1	2.20E+02	2.342423	100cp14Rep	8.00E+01	1.90309	Stnd3	4.50E+01	1.653213
	1.80E+02	2.255273		1.30E+02	2.113943		3.70E+01	1.568202
	2.40E+02	2.380211		6.30E+01	1.799341		4.90E+01	1.690196
	2.30E+02	2.361728		6.90E+01	1.838849		4.80E+01	1.681241
	2.40E+02	2.380211		6.60E+01	1.819544		5.00E+01	1.69897
	2.30E+02	2.361728		7.70E+01	1.886491		4.60E+01	1.662758
	3.80E+02	2.579784		5.90E+01	1.770852		8.10E+01	1.908485
	2.40E+02	2.380211		7.70E+01	1.886491		5.00E+01	1.69897
	1.90E+02	2.278754		7.50E+01	1.875061		3.90E+01	1.591065
	5.40E+02	2.732394		8.00E+01	1.90309		1.20E+02	2.079181
	2.10E+02	2.322219		1.90E+02	2.278754		4.30E+01	1.633468
	3.90E+02	2.591065		1.30E+02	2.113943		8.40E+01	1.924279
mean	2.25E+02	2.329019	mean	7.43E+01	1.844398	mean	4.64E+01	1.638876
SD	80.54303	0.140982	SD	28.61324	0.150792	SD	18.32163	0.153999

Overall Log SD (across all three standard curves): 0.006782

Table S4.0.2. Overall Log difference in the high copy (HC) variations between standard curves

stnd1	HC (100000)		Stnd2(6/7/12)	HC (100000)		stnd3	HC (100000)	
Date	CN	Log	Date	CN	Log	Date	CN	Log
4/23/2012	1.00E+05	5.00E+00	4/23/2012	5.70E+04	4.76E+00	4/23/2012	3.70E+04	4.57E+00
	1.70E+05	5.23E+00		9.70E+04	4.99E+00		6.40E+04	4.81E+00
	1.40E+05	5.15E+00		7.90E+04	4.90E+00		5.20E+04	4.72E+00
	2.00E+05	5.30E+00		1.10E+05	5.04E+00		7.40E+04	4.87E+00
	8.00E+04	4.90E+00		4.20E+04	4.62E+00		2.80E+04	4.45E+00
	1.10E+05	5.04E+00		6.30E+04	4.80E+00		4.10E+04	4.61E+00
	8.40E+04	4.92E+00		4.50E+04	4.65E+00		2.90E+04	4.46E+00
	8.10E+04	4.91E+00		4.30E+04	4.63E+00		2.80E+04	4.45E+00
	8.90E+04	4.95E+00		4.80E+04	4.68E+00		3.10E+04	4.49E+00
	1.00E+05	5.00E+00		5.60E+04	4.75E+00		3.60E+04	4.56E+00
	6.10E+04	4.79E+00		3.20E+04	4.51E+00		2.10E+04	4.32E+00
	8.90E+04	4.95E+00		4.80E+04	4.68E+00		3.10E+04	4.49E+00
	8.70E+04	4.94E+00		4.70E+04	4.67E+00		3.00E+04	4.48E+00
5/18/2012	1.40E+05	5.15E+00	5/18/2012	7.90E+04	4.90E+00	5/18/2012	5.00E+04	4.70E+00
	1.50E+05	5.18E+00		8.50E+04	4.93E+00		5.20E+04	4.72E+00
	9.30E+04	4.97E+00		5.00E+04	4.70E+00		5.60E+04	4.75E+00
	1.60E+05	5.20E+00		8.90E+04	4.95E+00		3.30E+04	4.52E+00
	1.50E+05	5.18E+00		8.40E+04	4.92E+00		5.80E+04	4.76E+00
	2.00E+05	5.30E+00		1.20E+05	5.08E+00		5.50E+04	4.74E+00
	1.10E+05	5.04E+00		6.20E+04	4.79E+00		7.70E+04	4.89E+00
	1.50E+05	5.18E+00		8.70E+04	4.94E+00		4.00E+04	4.60E+00
	1.30E+05	5.11E+00		7.30E+04	4.86E+00		5.70E+04	4.76E+00
	1.30E+05	5.11E+00		7.50E+04	4.88E+00		4.80E+04	4.68E+00
	2.00E+05	5.30E+00		1.10E+05	5.04E+00		4.90E+04	4.69E+00
	1.40E+05	5.15E+00		7.80E+04	4.89E+00		7.30E+04	4.86E+00
	2.10E+05	5.32E+00		1.20E+05	5.08E+00		5.10E+04	4.71E+00
	1.40E+05	5.15E+00		8.00E+04	4.90E+00		7.80E+04	4.89E+00
	1.70E+05	5.23E+00		9.60E+04	4.98E+00		5.20E+04	4.72E+00
	1.40E+05	5.15E+00		8.10E+04	4.91E+00		6.30E+04	4.80E+00
	2.20E+05	5.34E+00		1.30E+05	5.11E+00		5.30E+04	4.72E+00
	1.30E+05	5.11E+00		7.30E+04	4.86E+00		8.20E+04	4.91E+00
	1.70E+05	5.23E+00		9.80E+04	4.99E+00		4.80E+04	4.68E+00
	2.70E+05	5.43E+00		1.60E+05	5.20E+00		6.40E+04	4.81E+00
	1.90E+05	5.28E+00		1.10E+05	5.04E+00		1.00E+05	5.00E+00
	2.50E+05	5.40E+00		1.50E+05	5.18E+00		7.00E+04	4.85E+00
	1.60E+05	5.20E+00		8.80E+04	4.94E+00		9.60E+04	4.98E+00
	1.50E+05	5.18E+00		8.30E+04	4.92E+00		5.70E+04	4.76E+00
	2.70E+05	5.43E+00		1.60E+05	5.20E+00		5.40E+04	4.73E+00
	2.30E+05	5.36E+00		1.40E+05	5.15E+00		1.00E+05	5.00E+00
	1.90E+05	5.28E+00		1.10E+05	5.04E+00		9.00E+04	4.95E+00
	3.10E+05	5.49E+00		1.90E+05	5.28E+00		7.00E+04	4.85E+00
	1.50E+05	5.18E+00		8.40E+04	4.92E+00		1.20E+05	5.08E+00
	3.70E+04	4.57E+00		1.40E+05	5.15E+00		5.50E+04	4.74E+00
mean	1.52E+05	5.15E+00	mean	8.96E+04	4.92E+00	mean	5.70E+04	4.72E+00
SD	59863.8174	1.86E-01	SD	36419.6654	1.80E-01	SD	22353.1754	1.73E-01

Overall Log SD across all three standard curves: **0.006186**

Table S4.0.3. Overall Log difference in the low copy (LC) variations between standard curve

stnd1	LC (400)		stnd2(6/7/12)	LC (400)		stnd3	LC (400)	
Date	CN	Log	Date	CN	Log	Date	CN	Log
4/23/2012	7.80E+02	2.892095	4/23/2012	2.20E+02	2.342423	4/23/2012	1.80E+02	2.255273
	3.30E+02	2.518514		2.40E+02	2.380211		7.00E+01	1.845098
	4.00E+02	2.60206		2.10E+02	2.322219		8.50E+01	1.929419
	4.20E+02	2.623249		2.80E+02	2.447158		8.90E+01	1.94939
	4.70E+02	2.672098		1.10E+02	2.041393		1.00E+02	2
	3.20E+02	2.50515		1.40E+02	2.146128		6.70E+01	1.826075
	4.00E+02	2.60206		1.40E+02	2.146128		8.50E+01	1.929419
	3.30E+02	2.518514		1.60E+02	2.20412		7.10E+01	1.851258
	3.90E+02	2.591065		1.10E+02	2.041393		8.40E+01	1.924279
	7.10E+02	2.851258		1.40E+02	2.146128		1.60E+02	2.20412
	6.20E+02	2.792392		1.10E+02	2.041393		1.40E+02	2.146128
	6.70E+02	2.826075		1.30E+02	2.113943		1.50E+02	2.176091
	5.90E+02	2.770852		2.60E+02	2.414973		1.30E+02	2.113943
4/25/2012	7.70E+02	2.886491	4/25/2012	2.80E+02	2.447158	4/25/2012	1.80E+02	2.255273
	4.20E+02	2.623249		1.50E+02	2.176091		9.10E+01	1.959041
	4.50E+02	2.653213		1.50E+02	2.176091		9.60E+01	1.982271
5/18/2012	9.20E+02	2.963788	5/18/2012	3.10E+02	2.491362	5/18/2012	2.10E+02	2.322219
stnd1	7.30E+02	2.863323		2.40E+02	2.380211		1.60E+02	2.20412
	5.00E+02	2.69897		1.60E+02	2.20412		1.10E+02	2.041393
	1.00E+03	3		3.60E+02	2.556303		2.40E+02	2.380211
	6.60E+02	2.819544		2.20E+02	2.342423		1.50E+02	2.176091
5/24/2012	9.10E+02	2.959041	5/24/2012	3.10E+02	2.491362	5/24/2012	2.10E+02	2.322219
	7.60E+02	2.880814		3.00E+02	2.477121		1.70E+02	2.230449
	1.10E+03	3.041393		2.30E+02	2.361728		2.50E+02	2.39794
	7.00E+02	2.845098		3.60E+02	2.556303		1.60E+02	2.20412
	9.40E+02	2.973128		2.20E+02	2.342423		2.20E+02	2.342423
	5.90E+02	2.770852		2.90E+02	2.462398		1.30E+02	2.113943
	1.60E+03	3.20412		4.00E+02	2.60206		3.90E+02	2.591065
	8.50E+02	2.929419		3.70E+02	2.568202		1.90E+02	2.278754
	9.90E+02	2.995635		2.70E+02	2.431364		2.30E+02	2.361728
	1.10E+03	3.041393		3.40E+02	2.531479		2.50E+02	2.39794
	8.50E+02	2.929419		3.90E+02	2.591065		2.00E+02	2.30103
	8.20E+02	2.913814		3.50E+02	2.544068		1.90E+02	2.278754
	6.20E+02	2.792392		2.10E+02	2.322219		1.40E+02	2.146128
	1.00E+03	3		6.30E+02	2.799341		2.30E+02	2.361728
	5.90E+02	2.770852		3.10E+02	2.491362		1.30E+02	2.113943
	6.30E+02	2.799341		2.10E+02	2.322219		1.40E+02	2.146128
	8.00E+02	2.90309		5.00E+02	2.69897		1.80E+02	2.255273
	7.60E+02	2.880814		2.70E+02	2.431364		1.70E+02	2.230449
	1.30E+03	3.113943		2.50E+02	2.39794		3.10E+02	2.491362
mean	7.20E+02	2.825463	mean	2.58E+02	2.374609	mean	1.63E+02	2.175912
SD	279.21582	0.170171	SD	1.10E+02	0.184353	SD	69.155696	0.18515

Overall Log SD across all the three standard curves: **0.0084**

Table S4.1.1 LOD was determined by replication experiments performed by different dates (CN – Copy Number per mL; LOD – Limit of Detection; SD – standard deviation).

Standard Curve 1

stnd1	LC (400)		stnd2(6/7/12)	LC (400)		stnd3	LC (400)	
Date	CN	Log	Date	CN	Log	Date	CN	Log
4/23/2012	7.80E+02	2.892095	4/23/2012	2.20E+02	2.342423	4/23/2012	1.80E+02	2.255273
	3.30E+02	2.518514		2.40E+02	2.380211		7.00E+01	1.845098
	4.00E+02	2.60206		2.10E+02	2.322219		8.50E+01	1.929419
	4.20E+02	2.623249		2.80E+02	2.447158		8.90E+01	1.94939
	4.70E+02	2.672098		1.10E+02	2.041393		1.00E+02	2
	3.20E+02	2.50515		1.40E+02	2.146128		6.70E+01	1.826075
	4.00E+02	2.60206		1.40E+02	2.146128		8.50E+01	1.929419
	3.30E+02	2.518514		1.60E+02	2.20412		7.10E+01	1.851258
	3.90E+02	2.591065		1.10E+02	2.041393		8.40E+01	1.924279
	7.10E+02	2.851258		1.40E+02	2.146128		1.60E+02	2.20412
	6.20E+02	2.792392		1.10E+02	2.041393		1.40E+02	2.146128
	6.70E+02	2.826075		1.30E+02	2.113943		1.50E+02	2.176091
	5.90E+02	2.770852		2.60E+02	2.414973		1.30E+02	2.113943
4/25/2012	7.70E+02	2.886491	4/25/2012	2.80E+02	2.447158	4/25/2012	1.80E+02	2.255273
	4.20E+02	2.623249		1.50E+02	2.176091		9.10E+01	1.959041
	4.50E+02	2.653213		1.50E+02	2.176091		9.60E+01	1.982271
5/18/2012	9.20E+02	2.963788	5/18/2012	3.10E+02	2.491362	5/18/2012	2.10E+02	2.322219
stnd1	7.30E+02	2.863323		2.40E+02	2.380211		1.60E+02	2.20412
	5.00E+02	2.69897		1.60E+02	2.20412		1.10E+02	2.041393
	1.00E+03	3		3.60E+02	2.556303		2.40E+02	2.380211
	6.60E+02	2.819544		2.20E+02	2.342423		1.50E+02	2.176091
5/24/2012	9.10E+02	2.959041	5/24/2012	3.10E+02	2.491362	5/24/2012	2.10E+02	2.322219
	7.60E+02	2.880814		3.00E+02	2.477121		1.70E+02	2.230449
	1.10E+03	3.041393		2.30E+02	2.361728		2.50E+02	2.39794
	7.00E+02	2.845098		3.60E+02	2.556303		1.60E+02	2.20412
	9.40E+02	2.973128		2.20E+02	2.342423		2.20E+02	2.342423
	5.90E+02	2.770852		2.90E+02	2.462398		1.30E+02	2.113943
	1.60E+03	3.20412		4.00E+02	2.60206		3.90E+02	2.591065
	8.50E+02	2.929419		3.70E+02	2.568202		1.90E+02	2.278754
	9.90E+02	2.995635		2.70E+02	2.431364		2.30E+02	2.361728
	1.10E+03	3.041393		3.40E+02	2.531479		2.50E+02	2.39794
	8.50E+02	2.929419		3.90E+02	2.591065		2.00E+02	2.30103
	8.20E+02	2.913814		3.50E+02	2.544068		1.90E+02	2.278754
	6.20E+02	2.792392		2.10E+02	2.322219		1.40E+02	2.146128
	1.00E+03	3		6.30E+02	2.799341		2.30E+02	2.361728
	5.90E+02	2.770852		3.10E+02	2.491362		1.30E+02	2.113943
	6.30E+02	2.799341		2.10E+02	2.322219		1.40E+02	2.146128
	8.00E+02	2.90309		5.00E+02	2.69897		1.80E+02	2.255273
	7.60E+02	2.880814		2.70E+02	2.431364		1.70E+02	2.230449
	1.30E+03	3.113943		2.50E+02	2.39794		3.10E+02	2.491362
mean	7.20E+02	2.825463	mean	2.58E+02	2.374609	mean	1.63E+02	2.175912
SD	279.21582	0.170171	SD	1.10E+02	0.184353	SD	69.155696	0.18515

Overall Log SD across all the three standard curves: **0.0084**

LOD (100)		
Date	CN	Log
**4/23/2012**	1.60E+02	2.20412
	2.00E+02	2.30103
stnd1	1.10E+02	2.041393
mean	1.57E+02	2.182181
SD	45.0925	0.131202

LOD (100)		
Date	CN	Log
**4/25/2012**	1.60E+02	2.20412
stnd1	1.10E+02	2.041393
100cp23Rep	2.10E+02	2.322219
	1.70E+02	2.230449
	2.40E+02	2.380211
	2.80E+02	2.447158
	1.60E+02	2.20412
	2.20E+02	2.342423
	2.20E+02	2.342423
	2.30E+02	2.361728
	2.90E+02	2.462398
	3.10E+02	2.491362
	1.40E+02	2.146128
	1.70E+02	2.230449
	2.00E+02	2.30103
	3.30E+02	2.518514
	1.90E+02	2.278754
	2.60E+02	2.414973
	2.10E+02	2.322219
	2.00E+02	2.30103
	2.10E+02	2.322219
	1.90E+02	2.278754
	1.10E+02	2.041393
mean	2.09E+02	2.303716
SD	57.91161	0.125593

LOD (100)		
Date	CN	Log
**5/24/2012**	2.00E+02	2.30103
100cp14Rep	2.30E+02	2.361728
Stnd1	2.20E+02	2.342423
	1.80E+02	2.255273
	2.40E+02	2.380211
	2.30E+02	2.361728
	2.40E+02	2.380211
	2.30E+02	2.361728
	3.80E+02	2.579784
	2.40E+02	2.380211
	1.90E+02	2.278754
	5.40E+02	2.732394
	2.10E+02	2.322219
	3.90E+02	2.591065
mean	2.66E+02	2.402054
SD	100.8208	0.135769

LC			LC			LC		
Date	CN	Log	Date	CN	Log	Date	CN	Log
**4/23/2012**	1.80E+02	2.255273	**5/18/2012**	2.10E+02	2.322219	**5/24/2012**	2.10E+02	2.322219
stnd3	7.00E+01	1.845098	stnd3	1.60E+02	2.20412	stnd3	1.70E+02	2.230449
400cp13Rep	8.50E+01	1.929419		1.10E+02	2.041393	400cp19Rep	2.50E+02	2.39794
	8.90E+01	1.94939		2.40E+02	2.380211		1.60E+02	2.20412
	1.00E+02	2		1.50E+02	2.1760		2.20E+02	2.342423

	6.70E+01	1.826075
	8.50E+01	1.929419
	7.10E+01	1.851258
	8.40E+01	1.924279
	1.60E+02	2.20412
	1.40E+02	2.146128
	1.50E+02	2.176091
	1.30E+02	2.113943
mean	1.09E+02	2.011576
SD	38.41574	0.148621

		91
mean	1.74E+02	2.224807
SD	51.28353	0.132365

**4/25/2012**	1.80E+02	2.255273
400cp3Rep	9.10E+01	1.959041
stnd3	9.60E+01	1.982271
mean	1.22E+02	2.065528
SD	50.00333	0.164733

	1.30E+02	2.113943
	3.90E+02	2.591065
	1.90E+02	2.278754
	2.30E+02	2.361728
	2.50E+02	2.39794
	2.00E+02	2.30103
	1.90E+02	2.278754
	1.40E+02	2.146128
	2.30E+02	2.361728
	1.30E+02	2.113943
	1.40E+02	2.146128
	1.80E+02	2.255273
	1.70E+02	2.230449
	3.10E+02	2.491362
mean	2.05E+02	2.292914
SD	65.01012	0.126456

Table S4.1.2 High-copy control (HC) was evaluated by replication experiments performed by different dates (The measurement of Day-to-day variations) (CN – Copy Number per mL; HC – High Copy number control; SD – standard deviation).

<u>Standard Curve 1</u> (file name: Stnd1-HC_CN-Log_day-to-day-variation.xlsx)

HC			HC		
Date	CN	Log	Date	CN	Log
**4/23/2012**	1.00E+05	5.00E+00	**5/18/2012**	1.40E+05	5.15E+00
(stnd1)	1.70E+05	5.23E+00	stnd1	1.50E+05	5.18E+00
13 Rep	1.40E+05	5.15E+00		9.30E+04	4.97E+00
	2.00E+05	5.30E+00		1.60E+05	5.20E+00
	8.00E+04	4.90E+00		1.50E+05	5.18E+00
	1.10E+05	5.04E+00		2.00E+05	5.30E+00
	8.40E+04	4.92E+00		1.10E+05	5.04E+00
	8.10E+04	4.91E+00		1.50E+05	5.18E+00
	8.90E+04	4.95E+00		1.30E+05	5.11E+00
	1.00E+05	5.00E+00		1.30E+05	5.11E+00
	6.10E+04	4.79E+00		2.00E+05	5.30E+00
	8.90E+04	4.95E+00		1.40E+05	5.15E+00
	8.70E+04	4.94E+00		2.10E+05	5.32E+00
mean	1.07E+05	5.01E+00		1.40E+05	5.15E+00
SD	39698.87	1.43E-01		1.70E+05	5.23E+00
				1.40E+05	5.15E+00
				2.20E+05	5.34E+00
				1.30E+05	5.11E+00
				1.70E+05	5.23E+00
				2.70E+05	5.43E+00
				1.90E+05	5.28E+00
				2.50E+05	5.40E+00
				1.60E+05	5.20E+00
				1.50E+05	5.18E+00
				2.70E+05	5.43E+00
				2.30E+05	5.36E+00
				1.90E+05	5.28E+00
				3.10E+05	5.49E+00
				1.50E+05	5.18E+00
				3.70E+04	4.57E+00
			mean	1.71E+05	5.21E+00
			SD	56952.69	1.70E-01

Standard Curve 2 (File: Stnd2-HC_CN-Log_day-to-day-variation.xlsx)

HC		
Date	CN	Log
**4/23/2012**	5.70E+04	4.76E+00
1E5 13Rep	9.70E+04	4.99E+00
Stnd2(6/7/12)	7.90E+04	4.90E+00
	1.10E+05	5.04E+00
	4.20E+04	4.62E+00
	6.30E+04	4.80E+00
	4.50E+04	4.65E+00
	4.30E+04	4.63E+00
	4.80E+04	4.68E+00
	5.60E+04	4.75E+00
	3.20E+04	4.51E+00
	4.80E+04	4.68E+00
	4.70E+04	4.67E+00
mean	5.90E+04	4.74E+00
SD	22967.37	1.53E-01

HC		
Date	CN	Log
**5/18/2012**	7.90E+04	4.90E+00
stnd2(6/7/12)	8.50E+04	4.93E+00
	5.00E+04	4.70E+00
	8.90E+04	4.95E+00
	8.40E+04	4.92E+00
	1.20E+05	5.08E+00
	6.20E+04	4.79E+00
	8.70E+04	4.94E+00
	7.30E+04	4.86E+00
	7.50E+04	4.88E+00
	1.10E+05	5.04E+00
	7.80E+04	4.89E+00
	1.20E+05	5.08E+00
	8.00E+04	4.90E+00
	9.60E+04	4.98E+00
	8.10E+04	4.91E+00
	1.30E+05	5.11E+00
	7.30E+04	4.86E+00
	9.80E+04	4.99E+00
	1.60E+05	5.20E+00
	1.10E+05	5.04E+00
	1.50E+05	5.18E+00
	8.80E+04	4.94E+00
	8.30E+04	4.92E+00
	1.60E+05	5.20E+00
	1.40E+05	5.15E+00
	1.10E+05	5.04E+00
	1.90E+05	5.28E+00
	8.40E+04	4.92E+00
	1.40E+05	5.15E+00
mean	1.03E+05	4.99E+00
SD	33193.36	1.34E-01

<u>Standard Curve 3 (File: Stnd3-HC_CN-Log_day-to-day-variation.xlsx)</u>

HC		
Date	CN	Log
**4/23/2012**	3.70E+04	4.57E+00
(stnd3)	6.40E+04	4.81E+00
1E5_13Rep	5.20E+04	4.72E+00
	7.40E+04	4.87E+00
	2.80E+04	4.45E+00
	4.10E+04	4.61E+00
	2.90E+04	4.46E+00
	2.80E+04	4.45E+00
	3.10E+04	4.49E+00
	3.60E+04	4.56E+00
	2.10E+04	4.32E+00
	3.10E+04	4.49E+00
	3.00E+04	4.48E+00
mean	3.86E+04	4.56E+00
SD	15569.94	1.56E-01

HC		
Date	CN	Log
**5/18/2012**	5.00E+04	4.70E+00
(stnd3)	5.20E+04	4.72E+00
	5.60E+04	4.75E+00
	3.30E+04	4.52E+00
	5.80E+04	4.76E+00
	5.50E+04	4.74E+00
	7.70E+04	4.89E+00
	4.00E+04	4.60E+00
	5.70E+04	4.76E+00
	4.80E+04	4.68E+00
	4.90E+04	4.69E+00
	7.30E+04	4.86E+00
	5.10E+04	4.71E+00
	7.80E+04	4.89E+00
	5.20E+04	4.72E+00
	6.30E+04	4.80E+00
	5.30E+04	4.72E+00
	8.20E+04	4.91E+00
	4.80E+04	4.68E+00
	6.40E+04	4.81E+00
	1.00E+05	5.00E+00
	7.00E+04	4.85E+00
	9.60E+04	4.98E+00
	5.70E+04	4.76E+00
	5.40E+04	4.73E+00
	1.00E+05	5.00E+00
	9.00E+04	4.95E+00
	7.00E+04	4.85E+00
	1.20E+05	5.08E+00
	5.50E+04	4.74E+00
mean	6.50E+04	4.79E+00
SD	20126.3	1.27E-01

Table S4.1.3 Low-copy control (LC) was evaluated with replication experiments performed by different dates (The measurement of Day-to-day variations) (CN – Copy Number per mL; LC – Low Copy number control; SD – standard deviation).

Standard Curve 1 (file name: Stnd1-LC_CN-Log_day-to-day-variation.xlsx)

LC		
Date	CN	Log
**4/23/2012**	7.80E+02	2.892095
(stnd1)	3.30E+02	2.518514
13 Rep	4.00E+02	2.60206
	4.20E+02	2.623249
	4.70E+02	2.672098
	3.20E+02	2.50515
	4.00E+02	2.60206
	3.30E+02	2.518514
	3.90E+02	2.591065
	7.10E+02	2.851258
	6.20E+02	2.792392
	6.70E+02	2.826075
	5.90E+02	2.770852
mean	4.95E+02	2.67426
SD	158.9348	0.13607

**4/25/2012**	7.70E+02	2.886491
(stnd1)	4.20E+02	2.623249
	4.50E+02	2.653213
mean	5.47E+02	2.720984
SD	193.9931	0.144114

LC		
Date	CN	Log
**5/18/2012**	9.20E+02	2.963788
stnd1	7.30E+02	2.863323
	5.00E+02	2.69897
	1.00E+03	3
	6.60E+02	2.819544
mean	7.62E+02	2.869125
SD	201.0473	0.119931

LC		
Date	CN	Log
**5/24/2012**	9.10E+02	2.959041
stnd1	7.60E+02	2.880814
400cp19Rep	1.10E+03	3.041393
	7.00E+02	2.845098
	9.40E+02	2.973128
	5.90E+02	2.770852
	1.60E+03	3.20412
	8.50E+02	2.929419
	9.90E+02	2.995635
	1.10E+03	3.041393
	8.50E+02	2.929419
	8.20E+02	2.913814
	6.20E+02	2.792392
	1.00E+03	3
	5.90E+02	2.770852
	6.30E+02	2.799341
	8.00E+02	2.90309
	7.60E+02	2.880814
	1.30E+03	3.113943
mean	8.90E+02	2.933924
SD	257.3368	0.116693

Standard Curve 2 (file name: Stnd2-LC_CN-Log_day-to-day-variation.xlsx)

HC		
Date	CN	Log
**4/23/2012**	5.70E+04	4.76E+00
1E5 13Rep	9.70E+04	4.99E+00
Stnd2(6/7/12)	7.90E+04	4.90E+00
	1.10E+05	5.04E+00
	4.20E+04	4.62E+00
	6.30E+04	4.80E+00
	4.50E+04	4.65E+00
	4.30E+04	4.63E+00
	4.80E+04	4.68E+00
	5.60E+04	4.75E+00
	3.20E+04	4.51E+00
	4.80E+04	4.68E+00
	4.70E+04	4.67E+00
mean	5.90E+04	4.74E+00
SD	22967.37	1.53E-01

HC		
Date	CN	Log
**5/18/2012**	7.90E+04	4.90E+00
stnd2(6/7/12)	8.50E+04	4.93E+00
	5.00E+04	4.70E+00
	8.90E+04	4.95E+00
	8.40E+04	4.92E+00
	1.20E+05	5.08E+00
	6.20E+04	4.79E+00
	8.70E+04	4.94E+00
	7.30E+04	4.86E+00
	7.50E+04	4.88E+00
	1.10E+05	5.04E+00
	7.80E+04	4.89E+00
	1.20E+05	5.08E+00
	8.00E+04	4.90E+00
	9.60E+04	4.98E+00
	8.10E+04	4.91E+00
	1.30E+05	5.11E+00
	7.30E+04	4.86E+00
	9.80E+04	4.99E+00
	1.60E+05	5.20E+00
	1.10E+05	5.04E+00
	1.50E+05	5.18E+00
	8.80E+04	4.94E+00
	8.30E+04	4.92E+00
	1.60E+05	5.20E+00
	1.40E+05	5.15E+00
	1.10E+05	5.04E+00
	1.90E+05	5.28E+00
	8.40E+04	4.92E+00
	1.40E+05	5.15E+00
mean	1.03E+05	4.99E+00
SD	33193.36	1.34E-01

Standard Curve 3 (file name: Stnd3-LC_CN-Log_day-to-day-variation.xlsx)

LC		
Date	CN	Log
**4/23/2012**	1.80E+02	2.255273
stnd3	7.00E+01	1.845098
400cp13Rep	8.50E+01	1.929419
	8.90E+01	1.94939
	1.00E+02	2
	6.70E+01	1.826075
	8.50E+01	1.929419
	7.10E+01	1.851258
	8.40E+01	1.924279
	1.60E+02	2.20412
	1.40E+02	2.146128
	1.50E+02	2.176091
	1.30E+02	2.113943
mean	1.09E+02	2.011576
SD	38.41574	0.148621

LC		
Date	CN	Log
**5/18/2012**	2.10E+02	2.322219
stnd3	1.60E+02	2.20412
	1.10E+02	2.041393
	2.40E+02	2.380211
	1.50E+02	2.176091
mean	1.74E+02	2.224807
SD	51.28353	0.132365

LC		
Date	CN	Log
**5/24/2012**	2.10E+02	2.322219
stnd3	1.70E+02	2.230449
400cp19Rep	2.50E+02	2.39794
	1.60E+02	2.20412
	2.20E+02	2.342423
	1.30E+02	2.113943
	3.90E+02	2.591065
	1.90E+02	2.278754
	2.30E+02	2.361728
	2.50E+02	2.39794
	2.00E+02	2.30103
	1.90E+02	2.278754
	1.40E+02	2.146128
	2.30E+02	2.361728
	1.30E+02	2.113943
	1.40E+02	2.146128
	1.80E+02	2.255273
	1.70E+02	2.230449
	3.10E+02	2.491362
mean	2.05E+02	2.292914
SD	65.01012	0.126456

**4/25/2012**	1.80E+02	2.255273
400cp3Rep	9.10E+01	1.959041
stnd3	9.60E+01	1.982271
mean	1.22E+02	2.065528
SD	50.00333	0.164733

Table S4.2.1 Replication experiments performed by different dates and different concentrations with the standards 1, 2, 3 (CN – Copy Number per mL; HC- high copy; LC – Low Copy number control; SD – standard deviation).

Standard Curve 1 (File name: CN_Log_HC-LC-LOD_40_Stnd1_vf.xlsx)

HC (100000)			LC (400)			LOD (100)		
Date	CN	Log	Date	CN	Log	Date	CN	Log
**4/23/2012**	1.00E+05	5.00E+00	**4/23/2012**	7.80E+02	2.892095	**4/23/2012**	1.60E+02	2.20412
(stnd1)	1.70E+05	5.23E+00	(stnd1)	3.30E+02	2.518514	400cp3Rep dil	2.00E+02	2.30103
13 Rep	1.40E+05	5.15E+00	13 Rep	4.00E+02	2.60206	stnd1	1.10E+02	2.041393
	2.00E+05	5.30E+00		4.20E+02	2.623249	**4/25/2012**	1.60E+02	2.20412
	8.00E+04	4.90E+00		4.70E+02	2.672098	stnd1	1.10E+02	2.041393
	1.10E+05	5.04E+00		3.20E+02	2.50515	100cp23Rep	2.10E+02	2.322219
	8.40E+04	4.92E+00		4.00E+02	2.60206		1.70E+02	2.230449
	8.10E+04	4.91E+00		3.30E+02	2.518514		2.40E+02	2.380211
	8.90E+04	4.95E+00		3.90E+02	2.591065		2.80E+02	2.447158
	1.00E+05	5.00E+00		7.10E+02	2.851258		1.60E+02	2.20412
	6.10E+04	4.79E+00		6.20E+02	2.792392		2.20E+02	2.342423
	8.90E+04	4.95E+00		6.70E+02	2.826075		2.20E+02	2.342423
	8.70E+04	4.94E+00		5.90E+02	2.770852		2.30E+02	2.361728
**5/18/2012**	1.40E+05	5.15E+00	**4/25/2012**	7.70E+02	2.886491		2.90E+02	2.462398
stnd1	1.50E+05	5.18E+00	(stnd1)	4.20E+02	2.623249		3.10E+02	2.491362
	9.30E+04	4.97E+00		4.50E+02	2.653213		1.40E+02	2.146128
	1.60E+05	5.20E+00	**5/18/2012**	9.20E+02	2.963788		1.70E+02	2.230449
	1.50E+05	5.18E+00	stnd1	7.30E+02	2.863323		2.00E+02	2.30103
	2.00E+05	5.30E+00		5.00E+02	2.69897		3.30E+02	2.518514
	1.10E+05	5.04E+00		1.00E+03	3		1.90E+02	2.278754
	1.50E+05	5.18E+00		6.60E+02	2.819544		2.60E+02	2.414973
	1.30E+05	5.11E+00	**5/24/2012**	9.10E+02	2.959041		2.10E+02	2.322219
	1.30E+05	5.11E+00	stnd1	7.60E+02	2.880814		2.00E+02	2.30103
	2.00E+05	5.30E+00	400cp19Rep	1.10E+03	3.041393		2.10E+02	2.322219
	1.40E+05	5.15E+00		7.00E+02	2.845098		1.90E+02	2.278754
	2.10E+05	5.32E+00		9.40E+02	2.973128		1.10E+02	2.041393
	1.40E+05	5.15E+00		5.90E+02	2.770852	**5/24/2012**	2.00E+02	2.30103
	1.70E+05	5.23E+00		1.60E+03	3.20412	100cp14Rep	2.30E+02	2.361728
	1.40E+05	5.15E+00		8.50E+02	2.929419	Stnd1	2.20E+02	2.342423
	2.20E+05	5.34E+00		9.90E+02	2.995635		1.80E+02	2.255273
	1.30E+05	5.11E+00		1.10E+03	3.041393		2.40E+02	2.380211
	1.70E+05	5.23E+00		8.50E+02	2.929419		2.30E+02	2.361728
	2.70E+05	5.43E+00		8.20E+02	2.913814		2.40E+02	2.380211
	1.90E+05	5.28E+00		6.20E+02	2.792392		2.30E+02	2.361728
	2.50E+05	5.40E+00		1.00E+03	3		3.80E+02	2.579784
	1.60E+05	5.20E+00		5.90E+02	2.770852		2.40E+02	2.380211
	1.50E+05	5.18E+00		6.30E+02	2.799341		1.90E+02	2.278754
	2.70E+05	5.43E+00		8.00E+02	2.90309		5.40E+02	2.732394

	2.30E+05	5.36E+00		7.60E+02	2.880814		2.10E+02	2.322219
	1.90E+05	5.28E+00		1.30E+03	3.113943		3.90E+02	2.591065
	3.10E+05	5.49E+00	mean	7.20E+02	2.825463	mean	2.25E+02	2.329019
	1.50E+05	5.18E+00	SD	279.2158	0.170171	SD	80.54303	0.140982
	3.70E+04	4.57E+00						
mean	1.52E+05	5.15E+00						
SD	59863.82	1.86E-01						

Standard Curve 2 (File name: CN_Log_HC-LC-LOD_40_Stnd2_vf.xlsx)

HC (100000)			LC (400)			LOD (100)		
Date	CN	Log	Date	CN	Log	Date	CN	Log
**4/23/2012**	5.70E+04	4.76E+00	**4/23/2012**	2.20E+02	2.342423	**4/23/2012**	5.50E+01	1.740363
1E5 13Rep	9.70E+04	4.99E+00	400cp13Rep	2.40E+02	2.380211	400cp3Rep dil	6.60E+01	1.819544
Stnd2(6/7/12)	7.90E+04	4.90E+00	stnd2(6/7/12)	2.10E+02	2.322219	stnd2(6/7/12)	3.60E+01	1.556303
	1.10E+05	5.04E+00		2.80E+02	2.447158	**4/25/2012**	6.70E+01	1.826075
	4.20E+04	4.62E+00		1.10E+02	2.041393	100cp23Rep	6.50E+01	1.812913
	6.30E+04	4.80E+00		1.40E+02	2.146128	stnd2(6/7/12)	5.50E+01	1.740363
	4.50E+04	4.65E+00		1.40E+02	2.146128		5.10E+01	1.70757
	4.30E+04	4.63E+00		1.60E+02	2.20412		3.30E+01	1.518514
	4.80E+04	4.68E+00		1.10E+02	2.041393		6.80E+01	1.832509
	5.60E+04	4.75E+00		1.40E+02	2.146128		7.80E+01	1.892095
	3.20E+04	4.51E+00		1.10E+02	2.041393		9.40E+01	1.973128
	4.80E+04	4.68E+00		1.30E+02	2.113943		5.20E+01	1.716003
	4.70E+04	4.67E+00		2.60E+02	2.414973		7.40E+01	1.869232
**5/18/2012**	7.90E+04	4.90E+00	**4/25/2012**	2.80E+02	2.447158		7.00E+01	1.845098
stnd2(6/7/12)	8.50E+04	4.93E+00	stnd2(6/7/12)	1.50E+02	2.176091		7.60E+01	1.880814
	5.00E+04	4.70E+00		1.50E+02	2.176091		9.80E+01	1.991226
	8.90E+04	4.95E+00	**5/18/2012**	3.10E+02	2.491362		1.00E+02	2
	8.40E+04	4.92E+00	stnd2(6/7/12)	2.40E+02	2.380211		4.30E+01	1.633468
	1.20E+05	5.08E+00		1.60E+02	2.20412		5.60E+01	1.748188
	6.20E+04	4.79E+00		3.60E+02	2.556303		6.50E+01	1.812913
	8.70E+04	4.94E+00		2.20E+02	2.342423		1.10E+02	2.041393
	7.30E+04	4.86E+00	**5/24/2012**	3.10E+02	2.491362		6.30E+01	1.799341
	7.50E+04	4.88E+00	stnd2(6/7/12)	3.00E+02	2.477121		8.80E+01	1.944483
	1.10E+05	5.04E+00	400cp19Rep	2.30E+02	2.361728		6.90E+01	1.838849
	7.80E+04	4.89E+00		3.60E+02	2.556303		6.00E+01	1.778151
	1.20E+05	5.08E+00		2.20E+02	2.342423		3.30E+01	1.518514
	8.00E+04	4.90E+00		2.90E+02	2.462398	**5/24/2012**	7.20E+01	1.857332
	9.60E+04	4.98E+00		4.00E+02	2.60206	stnd2(6/7/12)	7.80E+01	1.892095
	8.10E+04	4.91E+00		3.70E+02	2.568202	100cp14Rep	8.00E+01	1.90309
	1.30E+05	5.11E+00		2.70E+02	2.431364		1.30E+02	2.113943
	7.30E+04	4.86E+00		3.40E+02	2.531479		6.30E+01	1.799341
	9.80E+04	4.99E+00		3.90E+02	2.591065		6.90E+01	1.838849
	1.60E+05	5.20E+00		3.50E+02	2.544068		6.60E+01	1.819544
	1.10E+05	5.04E+00		2.10E+02	2.322219		7.70E+01	1.886491
	1.50E+05	5.18E+00		6.30E+02	2.799341		5.90E+01	1.770852
	8.80E+04	4.94E+00		3.10E+02	2.491362		7.70E+01	1.886491
	8.30E+04	4.92E+00		2.10E+02	2.322219		7.50E+01	1.875061
	1.60E+05	5.20E+00		5.00E+02	2.69897		8.00E+01	1.90309
	1.40E+05	5.15E+00		2.70E+02	2.431364		1.90E+02	2.278754
	1.10E+05	5.04E+00		2.50E+02	2.39794		1.30E+02	2.113943
	1.90E+05	5.28E+00	mean	2.58E+02	2.374609	mean	7.43E+01	1.844398
	8.40E+04	4.92E+00	SD	1.10E+02	0.184353	SD	28.61324	0.150792
	1.40E+05	5.15E+00						
mean	8.96E+04	4.92E+00						
SD	36419.67	1.80E-01						

Standard Curve 3 (File name: CN_Log_HC-LC-LOD_40_Stnd3_vf.xlsx)

HC (100000)			LC (400)			LOD (100)		
Date	CN	Log	Date	CN	Log	Date	CN	Log
**4/23/2012**	3.70E+04	4.57E+00	**4/23/2012**	1.80E+02	2.255273	**4/23/2012**	3.40E+01	1.531479
(stnd3)	6.40E+04	4.81E+00	stnd3	7.00E+01	1.845098	Stnd3	4.10E+01	1.612784
1E5_13Rep	5.20E+04	4.72E+00	400cp13Rep	8.50E+01	1.929419	400cp3Rep dil	2.20E+01	1.342423
	7.40E+04	4.87E+00		8.90E+01	1.94939	**4/25/2012**	3.40E+01	1.531479
	2.80E+04	4.45E+00		1.00E+02	2	100cp23Rep	3.20E+01	1.50515
	4.10E+04	4.61E+00		6.70E+01	1.826075	stnd3	2.00E+01	1.30103
	2.90E+04	4.46E+00		8.50E+01	1.929419		4.20E+01	1.623249
	2.80E+04	4.45E+00		7.10E+01	1.851258		4.80E+01	1.681241
	3.10E+04	4.49E+00		8.40E+01	1.924279		5.90E+01	1.770852
	3.60E+04	4.56E+00		1.60E+02	2.20412		3.20E+01	1.50515
	2.10E+04	4.32E+00		1.40E+02	2.146128		4.60E+01	1.662758
	3.10E+04	4.49E+00		1.50E+02	2.176091		4.40E+01	1.643453
	3.00E+04	4.48E+00		1.30E+02	2.113943		4.70E+01	1.672098
**5/18/2012**	5.00E+04	4.70E+00	**4/25/2012**	1.80E+02	2.255273		6.10E+01	1.78533
(stnd3)	5.20E+04	4.72E+00	400cp3Rep	9.10E+01	1.959041		6.50E+01	1.812913
	5.60E+04	4.75E+00	stnd3	9.60E+01	1.982271		2.60E+01	1.414973
	3.30E+04	4.52E+00	**5/18/2012**	2.10E+02	2.322219		3.50E+01	1.544068
	5.80E+04	4.76E+00	stnd3	1.60E+02	2.20412		4.00E+01	1.60206
	5.50E+04	4.74E+00		1.10E+02	2.041393		7.00E+01	1.845098
	7.70E+04	4.89E+00		2.40E+02	2.380211		3.90E+01	1.591065
	4.00E+04	4.60E+00		1.50E+02	2.176091		5.40E+01	1.732394
	5.70E+04	4.76E+00	**5/24/2012**	2.10E+02	2.322219		4.20E+01	1.623249
	4.80E+04	4.68E+00	stnd3	1.70E+02	2.230449		4.10E+01	1.612784
	4.90E+04	4.69E+00	400cp19Rep	2.50E+02	2.39794		4.30E+01	1.633468
	7.30E+04	4.86E+00		1.60E+02	2.20412		3.70E+01	1.568202
	5.10E+04	4.71E+00		2.20E+02	2.342423		2.10E+01	1.322219
	7.80E+04	4.89E+00		1.30E+02	2.113943	**5/24/2012**	4.10E+01	1.612784
	5.20E+04	4.72E+00		3.90E+02	2.591065	100cp14Rep	4.80E+01	1.681241
	6.30E+04	4.80E+00		1.90E+02	2.278754	Stnd3	4.50E+01	1.653213
	5.30E+04	4.72E+00		2.30E+02	2.361728		3.70E+01	1.568202
	8.20E+04	4.91E+00		2.50E+02	2.39794		4.90E+01	1.690196
	4.80E+04	4.68E+00		2.00E+02	2.30103		4.80E+01	1.681241
	6.40E+04	4.81E+00		1.90E+02	2.278754		5.00E+01	1.69897
	1.00E+05	5.00E+00		1.40E+02	2.146128		4.60E+01	1.662758
	7.00E+04	4.85E+00		2.30E+02	2.361728		8.10E+01	1.908485
	9.60E+04	4.98E+00		1.30E+02	2.113943		5.00E+01	1.69897
	5.70E+04	4.76E+00		1.40E+02	2.146128		3.90E+01	1.591065
	5.40E+04	4.73E+00		1.80E+02	2.255273		1.20E+02	2.079181
	1.00E+05	5.00E+00		1.70E+02	2.230449		4.30E+01	1.633468
	9.00E+04	4.95E+00		3.10E+02	2.491362		8.40E+01	1.924279
	7.00E+04	4.85E+00	mean	1.63E+02	2.175912	mean	4.64E+01	1.638876
	1.20E+05	5.08E+00	SD	69.1557	0.18515	SD	18.32163	0.153999
	5.50E+04	4.74E+00						
mean	5.70E+04	4.72E+00						
SD	22353.18	1.73E-01						

Table S4.2.4 (Figure S4.2.4) Quantitative real-time PCR of 50 patients' viral DNA was run side-by-side with calibrated DNA standard reagents for creating standard curve 1 (1E6, 1E5, 1E4, 1E3, 1E2) with controls of LC (low copy: LCA, LCA-B)), CAP (VLS9, V3, V4), NTC (water), IPC (HEX) within the same 96-well plate for intra-assay comparison.
(File name: 03-21-2012_stnd1-patients_CN_vf.xlsx)

Sample ID	Copy/mL
1.00E+05	1.00E+05
1.00E+05	1.00E+05
1.00E+05	1.00E+05
1129:Z0	6.70E+03
1201:Z00	2.20E+02
0108:Z000	8.40E+00
1124:Z0	3.50E+01
1125:Z0	1.40E+02
0131:Z00	2.70E+02
0206:Z000	3.50E+02
0109:Z00	3.00E+04
2-B	9.00E+01
1124:Z0	1.40E+02
0112:Z00	5.60E+04
0116:Z00	2.40E+03
1128:ZR	7.70E+01
0119:Z0R	6.90E+01
LCA-B	3.60E+02
1121:Z00023R	4.40E+01
VL510	1.90E+02
1201:Z11R	1.50E+02
1128:16R	1.90E+02
VLS9	1.50E+04
1127:05R	1.10E+02
1129:Z3R	9.10E+00
LCA	3.10E+02
LCA	4.70E+02
LCA	5.50E+02
0103:Z5R	1.00E+01
V3	1.90E+03
1128:41R	1.80E+02
0110:Z1S	2.20E+01
1201:ZR	N/A
1205:Z0R	3.90E+02
Z005R	9.00E+04
Z0015R	N/A
1201:Z7R	7.40E+01
0105:Z18R	5.10E+04
0106:Z35R	N/A
1205:Z5R	3.30E+02

1205:Z8R	9.40E+02
Water	N/A
1.00E+03	1.00E+03
1.00E+03	1.00E+03
1.00E+03	1.00E+03
1.00E+06	1.00E+06
1.00E+06	1.00E+06
1.00E+06	1.00E+06
1.00E+04	1.00E+04
1.00E+04	1.00E+04
1.00E+04	1.00E+04
1.00E+02	1.00E+02
1.00E+02	1.00E+02
1.00E+02	1.00E+02

Table S5.1 Replication experiments performed for determining the day-to-day variation and standard-to-standard variation across three different standard curves (standard curve 1, standard curve 2, and standard curve 3) (CN – Copy Number per mL; LOD – Limit of Detection; SD – standard deviation). (File name: Overall-Stnds-1-2-3_100cp_LOD_variation.xlsx)

Stnd1	LOD (100)		stnd2(6/7/12)	LOD (100)		Stnd3	LOD (100)	
Date	CN	Log	Date	CN	Log	Date	CN	Log
**4/23/2012**	1.60E+02	2.20412	**4/23/2012**	5.50E+01	1.740363	**4/23/2012**	3.40E+01	1.531479
	2.00E+02	2.30103		6.60E+01	1.819544		4.10E+01	1.612784
	1.10E+02	2.041393		3.60E+01	1.556303		2.20E+01	1.342423
**4/25/2012**	1.60E+02	2.20412	**4/25/2012**	6.70E+01	1.826075	**4/25/2012**	3.40E+01	1.531479
	1.10E+02	2.041393		6.50E+01	1.812913		3.20E+01	1.50515
	2.10E+02	2.322219		5.50E+01	1.740363		2.00E+01	1.30103
	1.70E+02	2.230449		5.10E+01	1.70757		4.20E+01	1.623249
	2.40E+02	2.380211		3.30E+01	1.518514		4.80E+01	1.681241
	2.80E+02	2.447158		6.80E+01	1.832509		5.90E+01	1.770852
	1.60E+02	2.20412		7.80E+01	1.892095		3.20E+01	1.50515
	2.20E+02	2.342423		9.40E+01	1.973128		4.60E+01	1.662758
	2.20E+02	2.342423		5.20E+01	1.716003		4.40E+01	1.643453
	2.30E+02	2.361728		7.40E+01	1.869232		4.70E+01	1.672098
	2.90E+02	2.462398		7.00E+01	1.845098		6.10E+01	1.78533

	3.10E+02	2.491362		7.60E+01	1.880814		6.50E+01	1.812913
	1.40E+02	2.146128		9.80E+01	1.991226		2.60E+01	1.414973
	1.70E+02	2.230449		1.00E+02	2		3.50E+01	1.544068
	2.00E+02	2.30103		4.30E+01	1.633468		4.00E+01	1.60206
	3.30E+02	2.518514		5.60E+01	1.748188		7.00E+01	1.845098
	1.90E+02	2.278754		6.50E+01	1.812913		3.90E+01	1.591065
	2.60E+02	2.414973		1.10E+02	2.041393		5.40E+01	1.732394
	2.10E+02	2.322219		6.30E+01	1.799341		4.20E+01	1.623249
	2.00E+02	2.30103		8.80E+01	1.944483		4.10E+01	1.612784
	2.10E+02	2.322219		6.90E+01	1.838849		4.30E+01	1.633468
	1.90E+02	2.278754		6.00E+01	1.778151		3.70E+01	1.568202
	1.10E+02	2.041393		3.30E+01	1.518514		2.10E+01	1.322219
**5/24/2012**	2.00E+02	2.30103	**5/24/2012**	7.20E+01	1.857332	**5/24/2012**	4.10E+01	1.612784
100cp14Rep	2.30E+02	2.361728	stnd2(6/7/12)	7.80E+01	1.892095	100cp14Rep	4.80E+01	1.681241
Stnd1	2.20E+02	2.342423	100cp14Rep	8.00E+01	1.90309	Stnd3	4.50E+01	1.653213
	1.80E+02	2.255273		1.30E+02	2.113943		3.70E+01	1.568202
	2.40E+02	2.380211		6.30E+01	1.799341		4.90E+01	1.690196
	2.30E+02	2.361728		6.90E+01	1.838849		4.80E+01	1.681241
	2.40E+02	2.380211		6.60E+01	1.819544		5.00E+01	1.69897
	2.30E+02	2.361728		7.70E+01	1.886491		4.60E+01	1.662758
	3.80E+02	2.579784		5.90E+01	1.770852		8.10E+01	1.908485
	2.40E+02	2.380211		7.70E+01	1.886491		5.00E+01	1.69897
	1.90E+02	2.278754		7.50E+01	1.875061		3.90E+01	1.591065
	5.40E+02	2.732394		8.00E+01	1.90309		1.20E+02	2.079181
	2.10E+02	2.322219		1.90E+02	2.278754		4.30E+01	1.633468
	3.90E+02	2.591065		1.30E+02	2.113943		8.40E+01	1.924279
mean	2.25E+02	2.329019	mean	7.43E+01	1.844398	mean	4.64E+01	1.638876

SD	80.54303	0.140982	SD	28.61324	0.150792	SD	18.32163	0.153999

Overall Log SD (across all three standard curves): 0.006782

Table S5.2 Replication experiments performed for determining the day-to-day variation and standard-to-standard variation across three different standard curves (standard curve 1, standard curve 2, and standard curve 3) (CN – Copy Number per mL; HC – High Copy number; SD – standard deviation). (File name: Overall-Stnds-1-2-3_HC_variation_1Column.xlsx)

stnd1	HC (100000)		Stnd2(6/7/12)	HC (100000)		stnd3	HC (100000)	
Date	CN	Log	Date	CN	Log	Date	CN	Log
**4/23/2012**	1.00E+05	5.00E+00	**4/23/2012**	5.70E+04	4.76E+00	**4/23/2012**	3.70E+04	4.57E+00
	1.70E+05	5.23E+00		9.70E+04	4.99E+00		6.40E+04	4.81E+00
	1.40E+05	5.15E+00		7.90E+04	4.90E+00		5.20E+04	4.72E+00
	2.00E+05	5.30E+00		1.10E+05	5.04E+00		7.40E+04	4.87E+00
	8.00E+04	4.90E+00		4.20E+04	4.62E+00		2.80E+04	4.45E+00
	1.10E+05	5.04E+00		6.30E+04	4.80E+00		4.10E+04	4.61E+00
	8.40E+04	4.92E+00		4.50E+04	4.65E+00		2.90E+04	4.46E+00
	8.10E+04	4.91E+00		4.30E+04	4.63E+00		2.80E+04	4.45E+00
	8.90E+04	4.95E+00		4.80E+04	4.68E+00		3.10E+04	4.49E+00
	1.00E+05	5.00E+00		5.60E+04	4.75E+00		3.60E+04	4.56E+00
	6.10E+04	4.79E+00		3.20E+04	4.51E+00		2.10E+04	4.32E+00
	8.90E+04	4.95E+00		4.80E+04	4.68E+00		3.10E+04	4.49E+00
	8.70E+04	4.94E+00		4.70E+04	4.67E+00		3.00E+04	4.48E+00
**5/18/2012**	1.40E+05	5.15E+00	**5/18/2012**	7.90E+04	4.90E+00	**5/18/2012**	5.00E+04	4.70E+00
	1.50E+05	5.18E+00		8.50E+04	4.93E+00		5.20E+04	4.72E+00
	9.30E+04	4.97E+00		5.00E+04	4.70E+00		5.60E+04	4.75E+00
	1.60E+05	5.20E+00		8.90E+04	4.95E+00		3.30E+04	4.52E+00
	1.50E+05	5.18E+00		8.40E+04	4.92E+00		5.80E+04	4.76E+00
	2.00E+05	5.30E+00		1.20E+05	5.08E+00		5.50E+04	4.74E+00
	1.10E+05	5.04E+00		6.20E+04	4.79E+00		7.70E+04	4.89E+00
	1.50E+05	5.18E+00		8.70E+04	4.94E+00		4.00E+04	4.60E+00
	1.30E+05	5.11E+00		7.30E+04	4.86E+00		5.70E+04	4.76E+00
	1.30E+05	5.11E+00		7.50E+04	4.88E+00		4.80E+04	4.68E+00
	2.00E+05	5.30E+00		1.10E+05	5.04E+00		4.90E+04	4.69E+00
	1.40E+05	5.15E+00		7.80E+04	4.89E+00		7.30E+04	4.86E+00
	2.10E+05	5.32E+00		1.20E+05	5.08E+00		5.10E+04	4.71E+00
	1.40E+05	5.15E+00		8.00E+04	4.90E+00		7.80E+04	4.89E+00
	1.70E+05	5.23E+00		9.60E+04	4.98E+00		5.20E+04	4.72E+00
	1.40E+05	5.15E+00		8.10E+04	4.91E+00		6.30E+04	4.80E+00
	2.20E+05	5.34E+00		1.30E+05	5.11E+00		5.30E+04	4.72E+00
	1.30E+05	5.11E+00		7.30E+04	4.86E+00		8.20E+04	4.91E+00
	1.70E+05	5.23E+00		9.80E+04	4.99E+00		4.80E+04	4.68E+00
	2.70E+05	5.43E+00		1.60E+05	5.20E+00		6.40E+04	4.81E+00
	1.90E+05	5.28E+00		1.10E+05	5.04E+00		1.00E+05	5.00E+00
	2.50E+05	5.40E+00		1.50E+05	5.18E+00		7.00E+04	4.85E+00

	1.60E+05	5.20E+00		8.80E+04	4.94E+00		9.60E+04	4.98E+00
	1.50E+05	5.18E+00		8.30E+04	4.92E+00		5.70E+04	4.76E+00
	2.70E+05	5.43E+00		1.60E+05	5.20E+00		5.40E+04	4.73E+00
	2.30E+05	5.36E+00		1.40E+05	5.15E+00		1.00E+05	5.00E+00
	1.90E+05	5.28E+00		1.10E+05	5.04E+00		9.00E+04	4.95E+00
	3.10E+05	5.49E+00		1.90E+05	5.28E+00		7.00E+04	4.85E+00
	1.50E+05	5.18E+00		8.40E+04	4.92E+00		1.20E+05	5.08E+00
	3.70E+04	4.57E+00		1.40E+05	5.15E+00		5.50E+04	4.74E+00
mean	1.52E+05	5.15E+00	mean	8.96E+04	4.92E+00	mean	5.70E+04	4.72E+00
SD	59863.82	1.86E-01	SD	36419.67	1.80E-01	SD	22353.18	1.73E-01

Overall Log SD across all three standard curves: **0.006186**

Table S5.3 Replication experiments performed for determining the day-to-day variation and standard-to-standard variation across three different standard curves (standard curve 1, standard curve 2, and standard curve 3) (CN – Copy Number per mL; LC – Low Copy number; SD – standard deviation) (File name: Overall-Stnds-1-2-3_LC_variation-1column.xlsx)

stnd1	LC (400)		stnd2(6/7/12)	LC (400)		stnd3	LC (400)	
Date	CN	Log	Date	CN	Log	Date	CN	Log
**4/23/2012**	7.80E+02	2.8920946	**4/23/2012**	2.20E+02	2.34242268	**4/23/2012**	1.80E+02	2.25527251
	3.30E+02	2.51851394		2.40E+02	2.38021124		7.00E+01	1.84509804
	4.00E+02	2.60205999		2.10E+02	2.32221929		8.50E+01	1.92941893
	4.20E+02	2.62324929		2.80E+02	2.44715803		8.90E+01	1.94939001
	4.70E+02	2.67209786		1.10E+02	2.04139269		1.00E+02	2
	3.20E+02	2.50514998		1.40E+02	2.14612804		6.70E+01	1.8260748
	4.00E+02	2.60205999		1.40E+02	2.14612804		8.50E+01	1.92941893
	3.30E+02	2.51851394		1.60E+02	2.20411998		7.10E+01	1.85125835
	3.90E+02	2.59106461		1.10E+02	2.04139269		8.40E+01	1.92427929
	7.10E+02	2.85125835		1.40E+02	2.14612804		1.60E+02	2.20411998
	6.20E+02	2.79239169		1.10E+02	2.04139269		1.40E+02	2.14612804
	6.70E+02	2.8260748		1.30E+02	2.11394335		1.50E+02	2.17609126
	5.90E+02	2.77085201		2.60E+02	2.41497335		1.30E+02	2.11394335
**4/25/2012**	7.70E+02	2.88649073	**4/25/2012**	2.80E+02	2.44715803	**4/25/2012**	1.80E+02	2.25527251
	4.20E+02	2.62324929		1.50E+02	2.17609126		9.10E+01	1.95904139
	4.50E+02	2.65321251		1.50E+02	2.17609126		9.60E+01	1.98227123
**5/18/2012**	9.20E+02	2.96378783	**5/18/2012**	3.10E+02	2.49136169	**5/18/2012**	2.10E+02	2.32221929
stnd1	7.30E+02	2.86332286		2.40E+02	2.38021124		1.60E+02	2.20411998
	5.00E+02	2.69897		1.60E+02	2.20411998		1.10E+02	2.04139269
	1.00E+03	3		3.60E+02	2.5563025		2.40E+02	2.38021124
	6.60E+02	2.81954394		2.20E+02	2.34242268		1.50E+02	2.17609126
**5/24/2012**	9.10E+02	2.95904139	**5/24/2012**	3.10E+02	2.49136169	**5/24/2012**	2.10E+02	2.32221929
	7.60E+02	2.88081359		3.00E+02	2.47712125		1.70E+02	2.23044892
	1.10E+03	3.04139269		2.30E+02	2.36172784		2.50E+02	2.39794001
	7.00E+02	2.84509804		3.60E+02	2.5563025		1.60E+02	2.20411998
	9.40E+02	2.97312785		2.20E+02	2.34242268		2.20E+02	2.34242268
	5.90E+02	2.77085201		2.90E+02	2.462398		1.30E+02	2.11394335
	1.60E+03	3.20411998		4.00E+02	2.60205999		3.90E+02	2.59106461

	8.50E+02	2.92941893		3.70E+02	2.56820172		1.90E+02	2.2787536
	9.90E+02	2.99563519		2.70E+02	2.43136376		2.30E+02	2.36172784
	1.10E+03	3.04139269		3.40E+02	2.53147892		2.50E+02	2.39794001
	8.50E+02	2.92941893		3.90E+02	2.59106461		2.00E+02	2.30103
	8.20E+02	2.91381385		3.50E+02	2.54406804		1.90E+02	2.2787536
	6.20E+02	2.79239169		2.10E+02	2.32221929		1.40E+02	2.14612804
	1.00E+03	3		6.30E+02	2.79934055		2.30E+02	2.36172784
	5.90E+02	2.77085201		3.10E+02	2.49136169		1.30E+02	2.11394335
	6.30E+02	2.79934055		2.10E+02	2.32221929		1.40E+02	2.14612804
	8.00E+02	2.90308999		5.00E+02	2.69897		1.80E+02	2.25527251
	7.60E+02	2.88081359		2.70E+02	2.43136376		1.70E+02	2.23044892
	1.30E+03	3.11394335		2.50E+02	2.39794001		3.10E+02	2.49136169
mean	7.20E+02	2.82546286	mean	2.58E+02	2.37460886	mean	1.63E+02	2.17591218
SD	279.21582	0.17017053	SD	1.10E+02	0.1843528	SD	69.1556958	0.18515036
**Overall Log SD across all the three standard curves:**					**0.00843**			

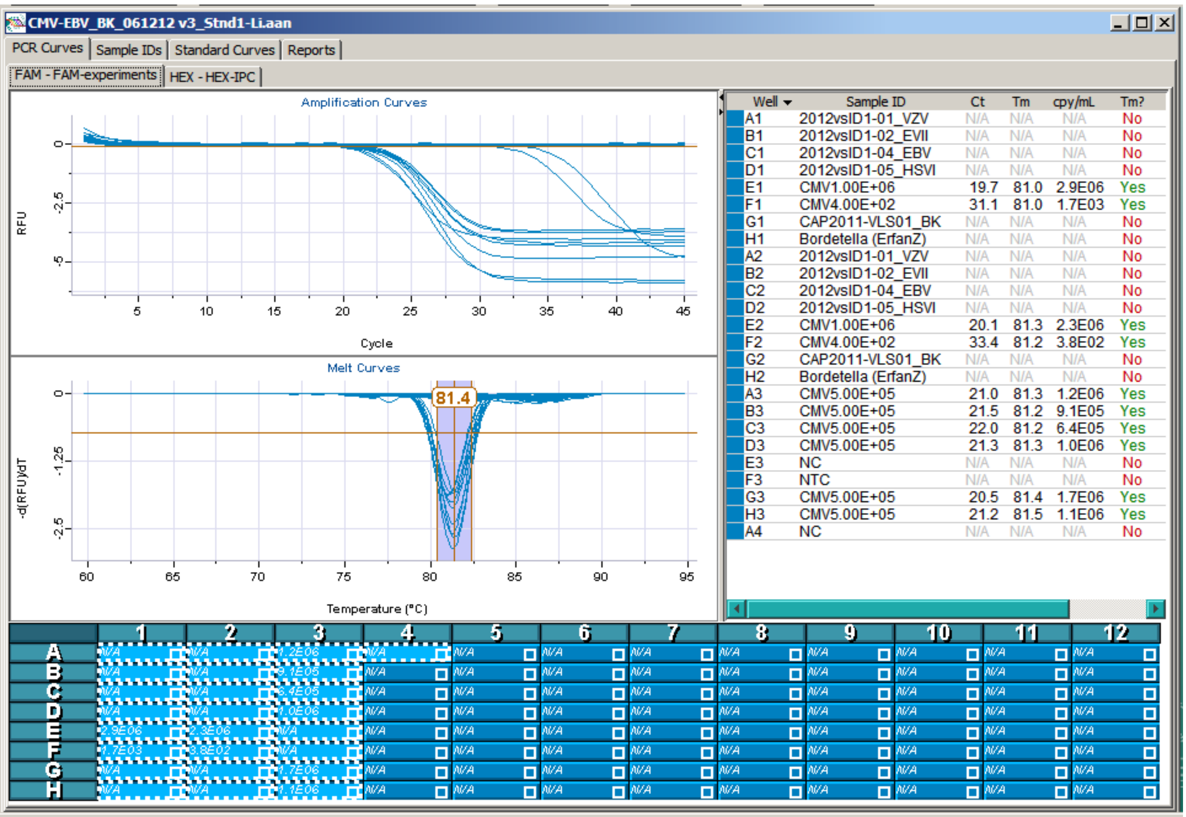

Figure S5. Analytical specificity was established first by different control virus sample studies of CMV, BK, EBV, EVII, HSVI, bordetella bacterial DNA (**quantitation with the standard curve 1**). "2012 viral survey ID1-01_VZV" was for Varicella-zoster virus. “2012_ID1-02_EVII” was positive for echovirus type II. "2012 viral survey ID1-04_EBV" was positive for Epstein-Barr virus. "2012vsID1-05_HSVI" was positive for Herpes simplex type I virus. "CAP2011-VLS01_BK" was positive for BK viral load (performed on June 12, 2012).

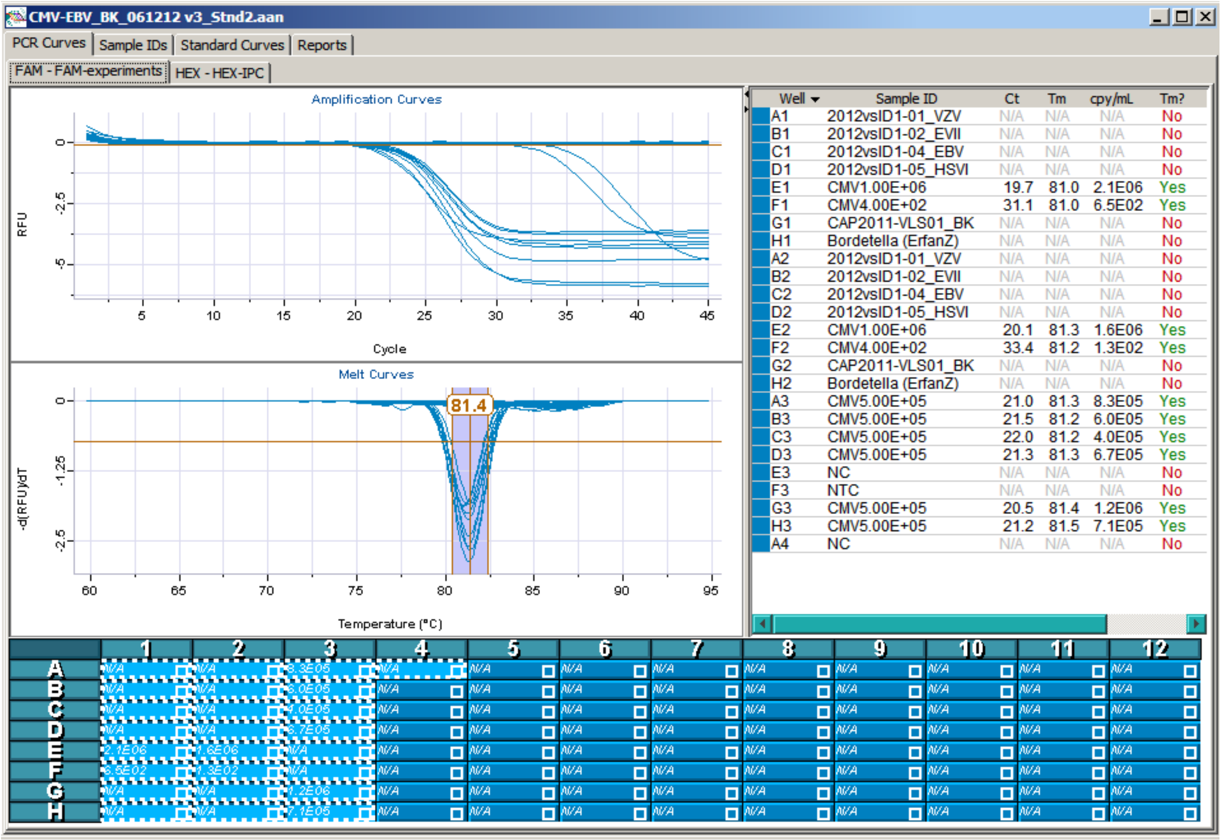

Figure S6. Analytical specificity was established first by different control virus sample studies of CMV, BK, EBV, EVII, HSVI, bordetella bacterial DNA (**quantitation with the standard curve 2**). "2012 viral survey ID1-01_VZV" was for Varicella-zoster virus. “2012_ID1-02_EVII” was positive for echovirus type II. "2012 viral survey ID1-04_EBV" was positive for Epstein-Barr virus. "2012vsID1-05_HSVI" was positive for Herpes simplex type I virus. "CAP2011-VLS01_BK" was positive for BK viral load (performed on June 12, 2012).

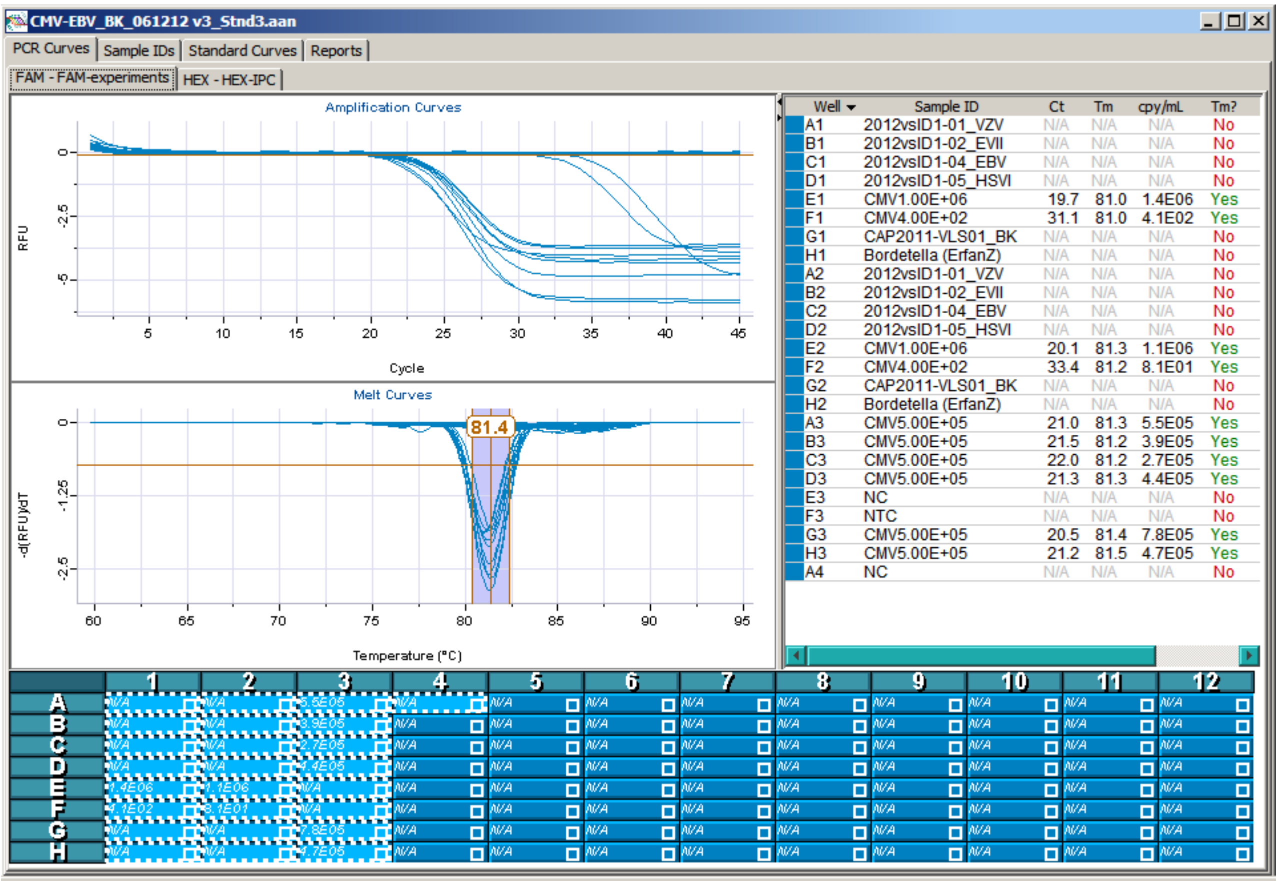

Figure S7. Analytical specificity was established first by different control virus sample studies of CMV, BK, EBV, EVII, HSVI, bordetella bacterial DNA (**quantitation with the standard curve 3**). "2012 viral survey ID1-01_VZV" was for Varicella-zoster virus. “2012_ID1-02_EVII” was positive for echovirus type II. "2012 viral survey ID1-04_EBV" was positive for Epstein-Barr virus. "2012vsID1-05_HSVI" was positive for Herpes simplex type I virus. "CAP2011-VLS01_BK" was positive for BK viral load (performed on June 12, 2012).

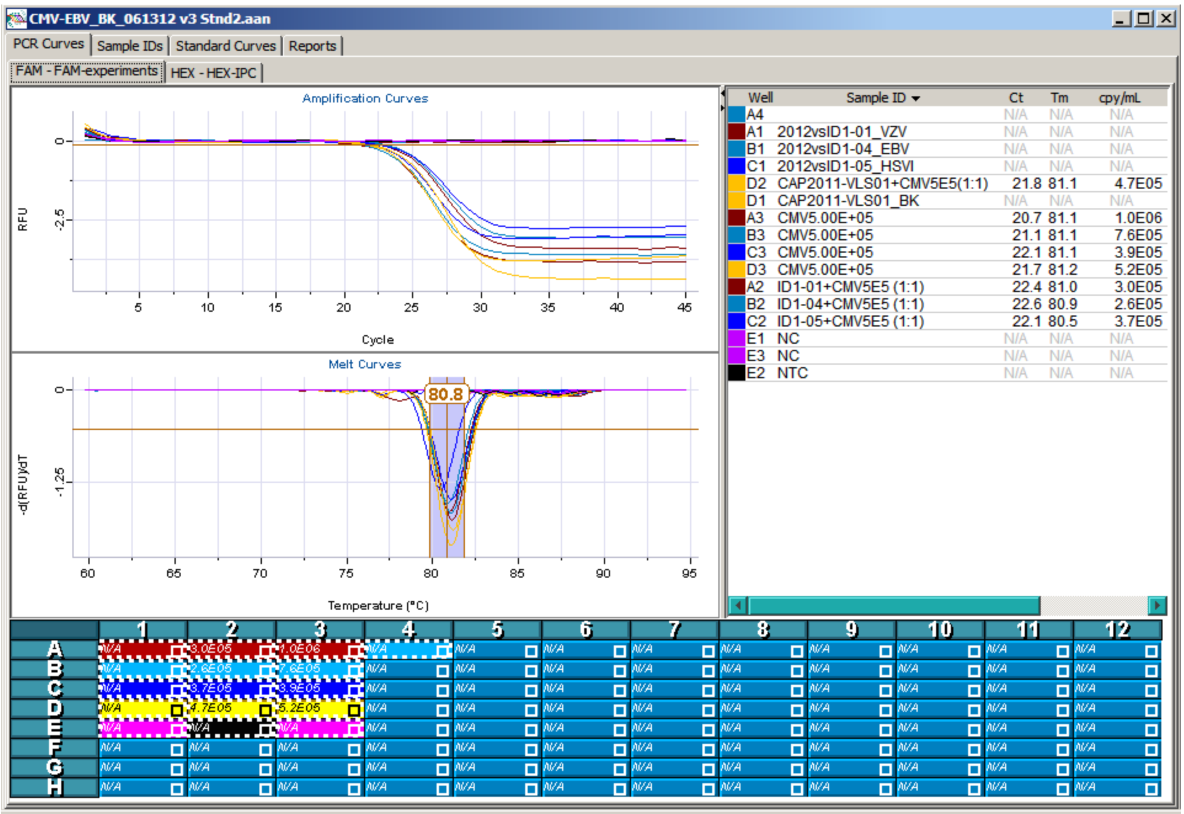

Figure S8. Analytical specificity was established secondly by interference study of CMV with BK, EBV, HSVI (quantitation with the standard curve 2). "2012 viral survey ID1-01_VZV" was for Varicella-zoster virus. "2012 viral survey ID1-04_EBV" was positive for Epstein-Barr virus. "2012vsID1-05_HSVI" was positive for Herpes simplex type I virus. "CAP2011-VLS01_BK" was positive for BK viral load.
Note: In all the mixtures, 2.5 micro-liter of CAP were mixed with 2.5 micro-liter of CMV 5E5 (5.00E+05) while in the controls either 5.0 micro-liter of CMV 5E5 (5.00E+05) or 5.0 micro-liter of CAP samples were used (performed on June 13, 2012).

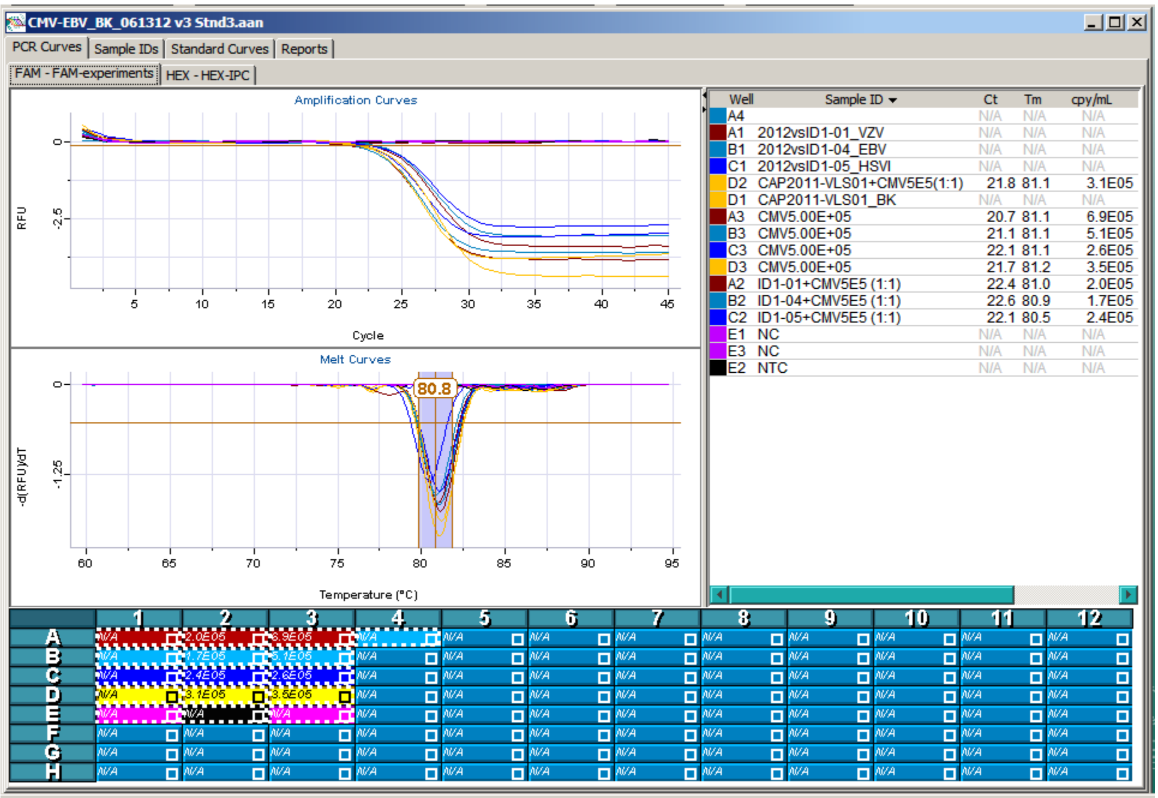

Figure S9. Analytical specificity was established second by interference study of CMV with BK, EBV, HSVI (quantitation with the standard curve 3). "2012 viral survey ID1-01_VZV" was for Varicella-zoster virus."2012 viral survey ID1-04_EBV" was positive for Epstein-Barr virus. "2012vsID1-05_HSVI" was positive for Herpes simplex type I virus. "CAP2011-VLS01_BK" was positive for BK viral load. Note: In all the mixtures, 2.5 micro-liter of CAP were mixed with 2.5 micro-liter of CMV 5E5 (5.00E+05) while in the controls either 5.0 micro-liter of CMV 5E5 (5.00E+05) or 5.0 micro-liter of CAP samples were used (performed on June 12, 2012).
